# The *brlA* Gene Deletion Reveals That Patulin Biosynthesis Is Not Related to Conidiation in *Penicillium expansum*

**DOI:** 10.3390/ijms21186660

**Published:** 2020-09-11

**Authors:** Chrystian Zetina-Serrano, Ophélie Rocher, Claire Naylies, Yannick Lippi, Isabelle P. Oswald, Sophie Lorber, Olivier Puel

**Affiliations:** Toxalim (Research Centre in Food Toxicology), Université de Toulouse, INRAE, ENVT, INP-Purpan, UPS, 31027 Toulouse, France; Chrystian-Del-Carmen.Zetina-Serrano@inrae.fr (C.Z.-S.); ophelie.rocher@inrae.fr (O.R.); claire.naylies@inrae.fr (C.N.); yannick.lippi@inrae.fr (Y.L.); isabelle.oswald@inrae.fr (I.P.O.); sophie.lorber@inrae.fr (S.L.)

**Keywords:** *Penicillium expansum*, *brlA*, conidiogenesis, synnemata, secondary metabolism, patulin, chaetoglobosins, communesins, metabolomics, microarray

## Abstract

Dissemination and survival of ascomycetes is through asexual spores. The *brlA* gene encodes a C_2_H_2_-type zinc-finger transcription factor, which is essential for asexual development. *Penicillium expansum* causes blue mold disease and is the main source of patulin, a mycotoxin that contaminates apple-based food. A *P. expansum* PeΔ*brlA* deficient strain was generated by homologous recombination. In vivo, suppression of *brlA* completely blocked the development of conidiophores that takes place after the formation of coremia/synnemata, a required step for the perforation of the apple epicarp. Metabolome analysis displayed that patulin production was enhanced by *brlA* suppression, explaining a higher in vivo aggressiveness compared to the wild type (WT) strain. No patulin was detected in the synnemata, suggesting that patulin biosynthesis stopped when the fungus exited the apple. In vitro transcriptome analysis of PeΔ*brlA* unveiled an up-regulated biosynthetic gene cluster (PEXP_073960-PEXP_074060) that shares high similarity with the chaetoglobosin gene cluster of *Chaetomium globosum*. Metabolome analysis of PeΔ*brlA* confirmed these observations by unveiling a greater diversity of chaetoglobosin derivatives. We observed that chaetoglobosins A and C were found only in the synnemata, located outside of the apple, whereas other chaetoglobosins were detected in apple flesh, suggesting a spatial-temporal organization of the chaetoglobosin biosynthesis pathway.

## 1. Introduction

*Penicillium* is a well-known genus of filamentous ascomycetous fungi. Taxonomically, it is a member of the *Aspergillaceae* family, in nature it is mainly found in the soil although it has also been detected in decaying organic matter, cereals, seeds, and various food products, and is consequently of great economic importance [1,2,3]. The genus currently contains 483 accepted species [3], fungi that are very common in the environment and are important in different fields including biotechnology, medical and food industries but also in phytopathology and food spoilage (pre- and postharvest pathogens) [4,5]. The species of this fungal genus are also known to produce biologically active compounds called secondary metabolites (SMs) that can range from potent pharmaceutical drugs to mycotoxins that are harmful to humans and animals [6,7,8,9,10].

*Penicillium expansum* is a post-harvest pathogen of apples and together with the species *Penicillium italicum* and *Penicillium digitatum* (citrus pathogens), it may cause up to 10% losses of harvested products [11]. *P. expansum* mainly infects apple fruit, but has also been isolated in other hosts including pears, cherries, peaches, plums, nuts, pecans, hazelnuts, and acorns [12,13,14,15]. *P. expansum* is a psychrophilic and necrotrophic fungus that develops during harvesting, postharvest processing, and storage through injuries that cause maceration and decomposition. It is considered to be the main agent of blue mold disease [16] and the main source of patulin in the human diet. Patulin is a toxic SM, undetectable by taste and smell, found not only in apple fruits but also in apple-based products. Heat treatments do not affect the overall stability of this mycotoxin and long-term exposure to patulin-contaminated products can cause serious health disorders. Patulin has been shown to be mutagenic, neurotoxic, genotoxic, cytotoxic, teratogenic, and immunotoxic to animals [15,17,18]. Due to its toxicity, maximum levels of patulin in food are regulated in most European countries (50, 25, and 10 μg of patulin/kg, in fruit juices, solid apple products, and apple-based products for infants, respectively) [19]. As for many fungal SMs, gene encoding enzymes, transporters and transcription factor (TF) involved in the biosynthesis of patulin are gathered into a cluster [11,20,21]. This cluster comprises 15 genes (PEXP_094320-PEXP_094460). Previous research has shown that this biosynthetic gene cluster is activated specifically by PatL (PEXP_094430) [11,21,22]. Patulin production is also positively regulated by PacC and CreA; these two TFs respond to abiotic stimuli such as pH and carbon source, respectively [23,24]. Some components of the velvet complex such as LaeA [25] and VeA [26] have been reported as positive regulators of the patulin biosynthesis. A recent study has shown that the deletion of *sntB*, a gene coding for an epigenetic reader, resulted in a decreased patulin production in vitro and *in planta* [27].

Although most studies on *P. expansum* have focused on patulin, the fungus produces many other SMs including citrinin, roquefortine C, chaetoglobosins A and C, expansolides A and B, andrastins A, B, and C and communesins [13]. The development of the fungus is generally linked to the production of SMs, [28,29,30,31] that may have specific ecological functions as virulence or aggressiveness factors, chemical weapons, communication signals, defense against fungivores or against damage [32].

*P. expansum* has a complex life cycle as its asexual life cycle involves four morphogenetic stages: (stage 1) vegetatively interconnected hyphal cells that form the mycelium, (stage 2) swelling of apical cells and subapical branching, (stage 3) formation of phialides, and (stage 4) formation of conidia [33]. The *brlA* (bristle), *abaA* (abacus-like), and *wetA* (wet-white) genes have been suggested to form a central regulatory pathway (CRP) that controls the expression of conidiation-specific genes [34,35,36]. The three genes are expressed sequentially and work in coordination to control the formation of conidiophores and conidium maturation [33,37,38]. The *abaA* gene is activated by BrlA in the middle stages of conidiophore development and is believed to be involved in the proper differentiation and functionality of phialides after the formation of metulae [34,36]. The *wetA* gene is activated by the *abaA* gene and is involved in the late stages of conidiation in the synthesis of crucial components (e.g., hydrophobins, melanins, and trehalose) of the cell wall layers that render mature conidia impermeable and resistant [39,40].

The *brlA* gene is expressed in the first stage of conidiation and encodes a C_2_H_2_-zinc-finger TF, which is considered a master regulator in the development of conidiophores. The structure of the *brlA* gene is complex and consists of two overlapping transcription units, *brlAα* and *brlAβ* [41]. These two transcription units are individually required for normal development but the products of these transcripts or mRNAs have redundant functions [42]. In the genus *Aspergillus*, the BrlA protein is mainly found in vesicles, metulae, and phialides but not in hyphae or mature conidia [37]. Studies of *Aspergillus nidulans* have shown that *brlA* is an extremely important gene in the CRP of conidiation since it activates the expression of *abaA* and *wetA* [41]. The *brlA* mutants have a “bristle-like” phenotype (no formation of conidia) because gene deletion blocks the transition of stalks to swollen vesicles and subsequent structures required for the formation of conidia resulting only in elongated aerial stalks [43,44]. On the other hand, overexpression of the *brlA* gene in vegetative cells leads to the formation of viable conidia directly from the tips of the hyphae [43,45]. In some fungi, including *Aspergillus clavatus* [45], *Aspergillus fumigatus* [31,46,47], and *Penicillium decumbens* [48], *brlA* has been shown to have an impact on the vegetative growth and biosynthesis of SMs, in addition to a major role in asexual reproduction, suggesting that *brlA* could have many more roles than those identified so far. Orthologs of *brlA* are only present in *Aspergillus* and *Penicillium* species [49,50,51,52].

To gain further insight into the functions of the *brlA* gene in *P. expansum*, a Pe∆*brlA* mutant was generated using the homologous recombination strategy. The impacts of the deletion of *brlA* on growth, in vitro macro and microscopic morphology, in vivo pathogenicity in apples, metabolome, and transcriptome (DNA microarray) were evaluated. Taken together, our data revealed that deletion of the *brlA* gene blocked conidiation but not the formation of synnemata formed by aggregation of hyphal mycelia. The ability to form synnemata was boosted when Pe∆*brlA* strain grew on cellulose medium under light-dark cycle compared to the WT strain. The *brlA* deletion impaired the typical *P. expansum* morphology and enhanced the in vivo aggressiveness. The production of the two best-known *P. expansum* mycotoxins patulin and citrinin was not impaired on synthetic media whereas a significantly increased production of patulin was observed in vivo, explaining the higher aggressiveness. The *brlA* deletion resulted in decreased communesin production. On other hand, an increase in chaetoglobosin biosynthesis was observed. The microarray analysis displayed a down-regulation of genes involved in communesin biosynthesis and it unveiled the up-regulation of an 11-gene cluster sharing high similarity with the chaetoglobosin gene cluster characterized in *Chaetomium globosum*.

## 2. Results

### 2.1. Effect of brlA Deletion on In Vitro Macroscopic and Microscopic Morphology

**Figure 1:**
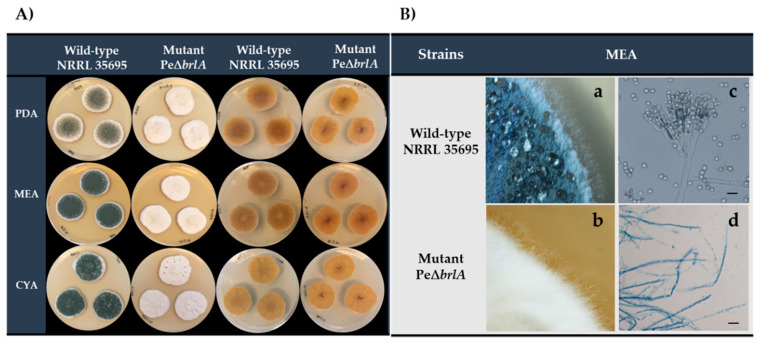
Morphological appearance of wild type *Penicillium expansum* and the null mutant Pe∆*brlA* strains. (**A**) Macroscopic appearance of the colonies (recto-verso). The strains were grown on Malt Extract agar (MEA), Potato Dextrose Agar (PDA), and Czapek Yeast extract Agar (CYA) for seven days at 25 °C in the dark. (**B**) Stereomicroscope observation (×12) after 10 days of incubation (**a**) wild type strain; (**b**) null mutant strain Pe∆*brlA*. Microscopic appearance (×400): (**c**) wild type conidiophores; (**d**) null mutant strain Pe∆*brlA* stalks after seven days of incubation. The strains were grown on MEA at 25 °C in the dark. Black scale bars represent 10 µm.

At the macroscopic level, the WT strain appeared blueish-green in the conidial areas with an external white margin, while the null mutant Pe∆*brlA* strain was entirely white throughout the colony, regardless of the culture media used (Figure 1A). As expected, the WT strain produced simple fused conidiophores (coremia) that emerged from the hyphae. The deletion of the BrlA TF-coding gene led to the complete absence of conidia but elongated hyphae stalks emerged, giving the null mutant strain a “bristle-like” appearance that was completely different from the velvety and granular texture of the WT strain (Figure 1Ba,b). The WT strain colony also displayed shallow radial furrows on Malt Extract agar (MEA) whereas they were not observed in the null mutant Pe∆*brlA* strain (Figure S1). Both strains produced droplets of exudate on the surface of the mycelium, but the WT strain seemed to produce more exudate than the null mutant Pe∆*brlA* strain. At a microscopic scale, the strains displayed completely different morphology (Figure 1Bc,d). The WT strain had terverticillate conidiophores, which branched from hyphae; phialides were cylindrical and conidia ellipsoid-circular (Figure 1Bc). Deletion of the *brlA* gene blocked asexual reproduction in *P. expansum*. The null mutant Pe∆*brlA* strain produced longer stalks but with no conidiophores, whose development was stopped before branches, metulae, and phialides were formed (Figure 1Bd).

### 2.2. Effect of brlA Deletion on Apple Colonization

#### 2.2.1. Effect of *brlA* Deletion on Pathogenicity

**Figure 2:**
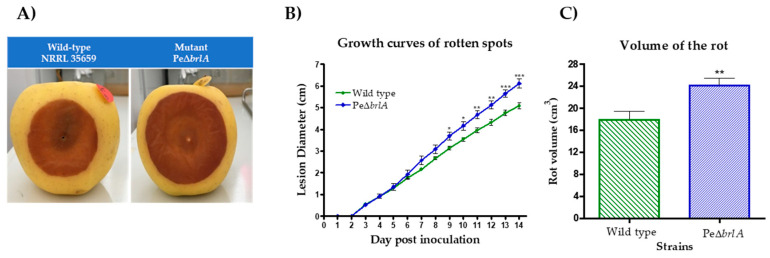
Golden Delicious apples infected with wild type *Penicillium expansum* or the null mutant Pe∆*brlA* strains, incubated at 25 °C for 14 days in the dark. (**A**) Spots of rot 11 days after infection. (**B**) Growth curves of spots. The diameter of the lesions was measured daily. (**C**) The volume of rot measured at the end of the 14-day incubation period using the method described by Baert et al. [16]. The graphs show the mean ± standard error of the mean (SEM) from nine biological replicates and the significant differences between the wild type and the null mutant Pe∆*brlA* strains. *p*-value * < 0.05; ** < 0.01; *** < 0.001.

To check if the null mutant Pe∆*brlA* strain could also trigger blue mold disease, Golden Delicious apples were infected. Both WT and null mutant strains were able to colonize the apples and showed the same development pattern for the first six days. An increase in rot diameter was observed in the null mutant PeΔ*brlA* strain from the seventh day and significant differences between the PeΔ*brlA* and WT strains were observed from the ninth day (Figure 2A,B). At the end of the 14-day incubation period, the diameter of the lesion caused by the PeΔ*brlA* strain was about 20% larger than the WT strain, 6.11 ± 0.14 cm and 5.08 ± 0.19 cm (*p*-value 7 × 10^−4^), respectively. The growth rates calculated from the growth curves proved that the rotting rate of the PeΔ*brlA* strain (0.50 ± 0.02 cm/day) was significantly higher than the WT strain (0.42 ± 0.01 cm/day) (*p*-value 5 × 10^−4^) (Figure S2). The rot volume calculated at the end of the incubation period showed that apples infected with the PeΔ*brlA* strain had a significantly higher rot volume than apples infected with the WT strain, 24.14 ± 1.31 cm^3^, and 17.95 ± 1.53 cm^3^ (*p*-value 7.2 × 10^−3^), respectively (Figure 2C).

#### 2.2.2. Effect of *brlA* Deletion on In Vivo Patulin Production

**Figure 3:**
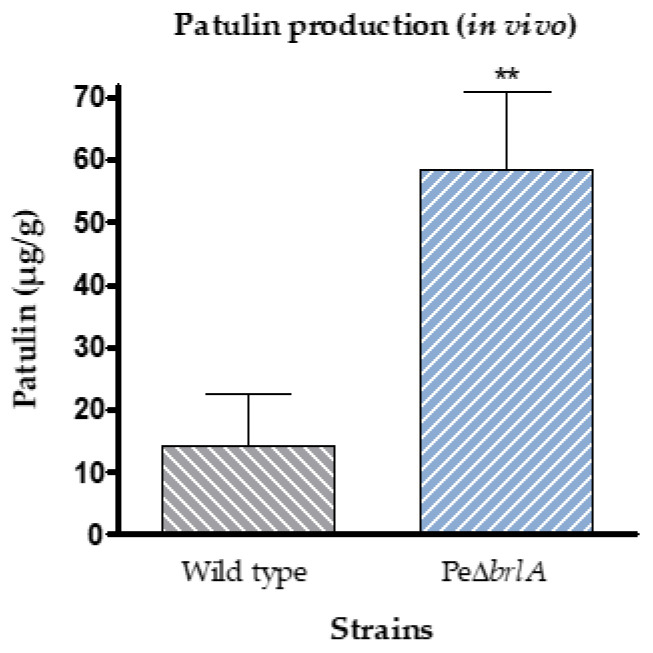
Patulin production in Golden Delicious apples infected with wild type *Penicillium expansum* or the null mutant Pe∆*brlA* strains at 14 days of incubation as previously described by Snini et al. [22]. Detection and quantification were performed by High Performance Liquid Chromatography-Diode Array Detector (HPLC-DAD) analysis at 277 nm and based on a standard curve, respectively. The graphs show the mean ± standard error of the mean (SEM) from nine biological replicates. *p*-value ** < 0.01.

Patulin concentrations measured in Golden Delicious apples after 14 days of incubation showed that the null mutant PeΔ*brlA* strain not only retained the ability to produce patulin, but produced four times the concentration produced by the WT strain, with 14.11 ± 8.3 µg/g and 58.5 ± 12.5 µg/g (*p*-value 9.3 × 10^−3^) fresh weight of apples, respectively (Figure 3).

#### 2.2.3. Effect of *brlA* Deletion on In Vivo Macroscopic Morphology

**Figure 4:**
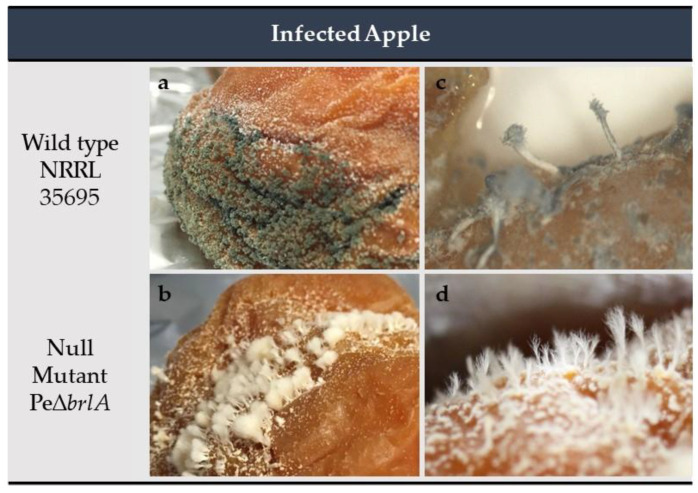
Apples infected with *Penicillium expansum* after 30 days of incubation at 25 °C in the dark. (**a**) Wild type strain; (**b**) Null mutant Pe∆*brlA* strain. Stereomicroscope observation (×12): (**c**) development of conidiophores in the wild type strain; (**d**) development of only sporeless synnemata in the null mutant Pe∆*brlA* strain. The experiment was carried out with four biological replicates.

After 30 days of development on Golden Delicious apples, results showed that the WT and Pe∆*brlA* strains completely invaded the apple mesocarp, causing tissue decay. When the fruit was fully colonized, the fungus drilled the apple epicarp and conidiogenesis occurred (Figure 4a). Normal asexual reproduction in the WT strain involves the production of conidiophores clustered in coremia (Figure 4a,c) whereas in the mutant Pe∆*brlA* strain, the conidiogenesis process was interrupted before the formation of metulae and only rigid, white, and sporeless hyphal structures (synnemata) developed (Figure 4b,d).

### 2.3. Growth Profile in Different Carbon Sources

**Figure 5:**
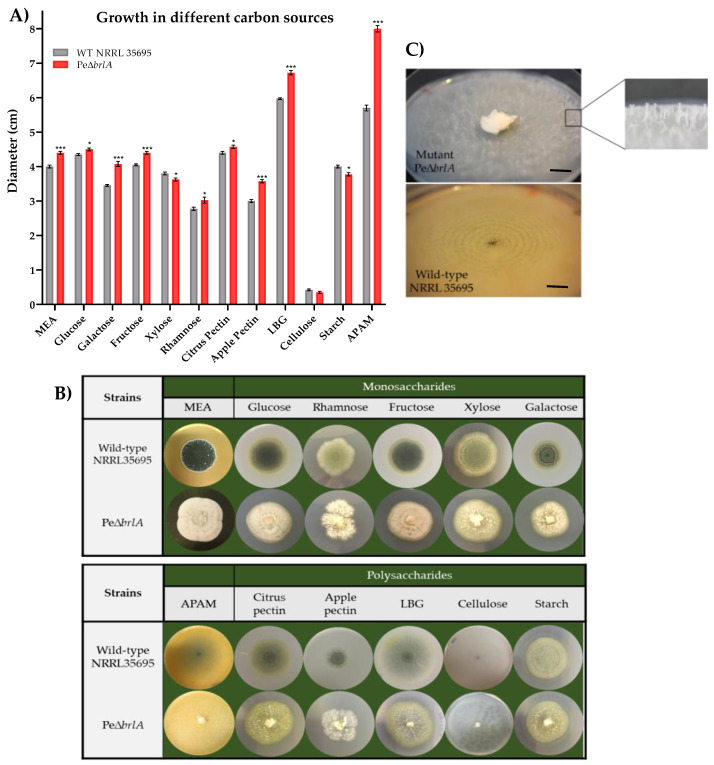
Wild type *Penicillium expansum* and the null mutant Pe∆*brlA* strains were grown in a minimal medium supplemented with different carbon sources for seven days at 25 °C in the dark. (**A**) Average diameter (cm) of the colonies and statistical analysis of the wild type and null mutant Pe∆*brlA* strains, developed in the different substrates. The graphs show the mean ± standard error of the mean (SEM) from three biological replicates and the significant differences between the wild type and the null mutant Pe∆*brlA* strains. *p*-value * < 0.05; *** < 0.001. (**B**) Photos of the strains cultured in monomeric or polymeric carbon sources. (**C**) Strains grown in cellulose-supplemented medium after 15 days of incubation at 25 °C in a 16:8 light/dark cycle (16L8D cycle). Black scale bars represent 10 mm.

The null mutant PeΔ*brlA* strain grew significantly more than the WT strain in 75% of cultures on minimal media supplemented with mono- or polysaccharides. Figure 5A shows the comparison of the fungal growth (colony diameters, in cm) between the WT and Pe∆*brlA* strains developed on different substrates.

Strain development was favored in glucose- and fructose-supplemented media, while rhamnose-enriched media produced the smallest diameters observed with values of 2.70 ± 0.04 cm and 3.05 ± 0.08 cm for the WT and PeΔ*brlA* strains, respectively. The difference in strain diameters was also apparent in the fructose- and galactose-enriched media where the diameters of the null mutant Pe∆*brlA* strain were up to 8.6% and 16% larger than the WT strain, respectively. Furthermore, carbon sources such as glucose, galactose, and fructose favored the growth of aerial mycelium in the null mutant PeΔ*brlA* strain (Figure 5B).

The polysaccharide locus bean gum (LBG) significantly favored the development of both strains, in contrast to cellulose in which very poor growth was observed. In starch- and cellulose-supplemented media the diameters of the mutant strain were smaller (−5% and −11.7%, respectively) than those of the WT strain. When the strains developed in the cellulose-supplemented medium using a 15-day incubation period and a 16:8 light/dark cycle (16L8D cycle), the diameter of the WT strain was 4.00 ± 0.02 cm, eight times the mean value obtained in a seven-day incubation period in the dark. The WT strain produced spores that formed concentric circles. Under the same growth conditions, the mutant PeΔ*brlA* strain increased its diameter value approximately 20 times (7.4 ± 0.1 cm) to the mean value obtained in a seven-day incubation period in the dark. The null mutant strain produced rigid synnemata that were scattered over the entire surface of the substrate (Figure 5C).

The PeΔ*brlA* strain showed significantly higher diameters than the WT strain in LBG and apple pectin-supplemented media with values of 12.5% and 19% higher, respectively. The diameters of both strains were bigger in the citrus pectin-supplemented medium than in the apple pectin-supplemented medium. However, the difference in diameter between the two strains was more pronounced when the strains were grown in apple pectin-enriched medium, the Pe∆*brlA* diameters were 19% larger than in the WT strain. The highest colony diameter values were obtained within the apple puree agar medium (APAM) [16], in which the diameter of the mutant PeΔ*brlA* strain was 40% larger than in the WT strain, similar to the in vivo results observed in apples.

Morphologically, the WT strain exhibited its characteristic blue-green color in almost all media, except in the rhamnose-, xylose-, and starch-supplement media, where its color was light green (Figure 5B). The mycelium of the null mutant PeΔ*brlA* strain was entirely white except in citrus pectin-, LBG- and starch-supplemented media, where the mycelium was slightly yellow in color (the origin of this yellow color was not determined). Glucose, fructose, and galactose favored the development of the aerial mycelium of the PeΔ*brlA* strain, giving it a fluffy texture (Figure S3a). Galactose favored the formation of coremia in the WT strain (Figure S3b) whereas in the rest of the substrates studied, this strain appeared to be flatter and smoother. The WT and null mutant strains produced no exudates in any of the media tested. Yellow halos were observed around both strains in the LBG-supplemented medium and in the WT strain in the citrus pectin-supplemented medium.

### 2.4. BrlA Is a Key Factor in the Regulation of Penicillium expansum Secondary Metabolites

#### 2.4.1. Secondary Metabolites Produced In Vitro

BrlA is a key regulator of fungal conidiation, but its role is not limited to asexual development. The ability to produce SMs by the null mutant Pe∆*brlA* strain was analyzed using liquid chromatography coupled with high-resolution mass spectrometry (LC-HRMS). The metabolites detected were identified after being cultured on labeled wheat and by comparing them with the reference metabolome of the WT strain grown under the same conditions. A total of 120 compounds were detected, of which 67 were identified and 53 remain unknown (Table 1). Some compounds with the same chemical formulae displayed similar MS/MS spectra (Tables S1 and S2).

The WT and Pe∆*brlA* strains have 65 metabolites (50%) in common while 23 were produced only by the WT strain and 32 are produced only in the null mutant strain. The production of patulin, citrinin, and roquefortines C and D was not affected by the deletion of the *brlA* gene. Intermediate compounds of the patulin biosynthetic pathway such as m-cresol, m-hydroxybenzyl alcohol, gentisyl alcohol, and ascladiol were also detected. The WT strain produced 12 communesin derivatives, whereas there was a drastic reduction in the production of these compounds in the null mutant strain, with only communesins I, E, Com470, A, D, and B represented. Moreover, aurantioclavine, an intermediate in the biosynthesis of communesins, was not detected, suggesting that it was not accumulated in the PeΔ*brlA* strain. Expansolides and andrastins A, B, and C were detected in both strains. Interestingly, the most common compounds found in the Pe∆*brlA* strain were the cytochalasans, of which 15 chaetoglobosins and one penochalasin were detected. In addition, a wide variety of unknown metabolites (18 compounds) were detected in the null mutant strain with chemical formulae C_32_H_38_N_2_O_4_ (R_T_ = 35.59, 37.93, and 38.15), C_32_H_38_N_2_O_5_ (R_T_ = 21.75, 22.76, 23.91, 27.38, and 28.52), C_32_H_36_N_2_O_6_ (R_T_ = 27.60, 29.23, 30.57, 31.50, 32.48, and 34.05), and C_32_H_38_N_2_O_6_ (R_T_ = 23.19, 25.47, 27.09, and 28.27). Comparison of MS/MS spectra with those of chaetoglobosins A and C suggested that the compounds are dehydroxylated and saturated (C_32_H_38_N_2_O_4_), saturated (C_32_H_38_N_2_O_5_), hydroxylated (C_32_H_36_N_2_O_6_), or saturated and hydroxylated (C_32_H_38_N_2_O_6_) forms of chaetoglobosin. Hence, there are 34 members of the cytochalasan alkaloid (chaetoglobosins/cytochalasins) family. Unknown metabolites (17) with chemical formulae C_17_H_23_N_3_O_3_ (R_T_ = 3.12 and 25.05), C_18_H_35_N_3_O_4_ (R_T_ = 3.54), C_19_H_20_O_5_ (R_T_ = 33.29), C_20_H_21_NO_9_ (R_T_ = 22.19), C_26_H_32_O_8_ (R_T_ = 9.67, 10.64, and 34.14), C_27_H_29_N_5_O_4_ (R_T_ = 10.80 and 11.03), C_29_H_27_N_5_O_5_ (R_T_ = 15.70 and 17.61), C_29_H_31_N_5_O_5_ (R_T_ = 14.83, 15.85, and 16.68), and C_29_H_33_N_5_O_6_ (R_T_ = 11.36 and 12.14) were found only in the null mutant Pe∆*brlA* strain. Unknown compounds (17) with chemical formulae C_10_H_17_NO_5_ (R_T_ = 9.65), C_15_H_20_O_4_ (R_T_ = 16.91), C_18_H_16_N_2_O_2_ (R_T_ = 13.58 and 17.74), C_19_H_16_N_2_O_2_ (R_T_ = 32.50 and 33.17), C_18_H_16_N_2_O_3_ (R_T_ = 14.76), C_20_H_18_N_2_O_2_ (R_T_ = 35.48 and 36.44), C_19_H_16_N_2_O_4_ (R_T_ = 13.73 and 15.20), C_19_H_38_O_6_ (R_T_ = 37.23 and 38.19), C_24_H_26_N_2_O_6_ (R_T_ = 33.79), C_28_H_32_N_4_O_3_ (R_T_ = 15.50), C_28_H_38_O_8_ (R_T_ = 28.44), and C_29_H_33_N_5_O_6_ (R_T_ = 15.42) were no longer produced in the null mutant strain.

#### 2.4.2. Secondary Metabolites Produced In Vivo

In vivo results revealed that after 30 days incubation, the null mutant Pe∆*brlA* strain had completely colonized the fruit. Table 2 lists the 33 compounds detected in apple flesh infected with the null mutant strain. Gentisyl alcohol and ascladiol were found in addition to the final product of patulin biosynthesis. Citrinin, expansolides A/B, roquefortine C, andrastins A and B were also identified. Nine chaetoglobosins and five putative members of the cytochalasan family were also detected. However, only one communesin derivative, communesin B, was found.

In the second step, the synnemata that developed on the apple epicarp were extracted and the SMs present were analyzed. Table 3 lists the 54 SMs detected in the synnemata from the null mutant Pe∆*brlA* strain. Patulin and citrinin were no longer produced in synnemata. However, 21 of the 34 members of the cytochalasan family, including the well-known chaetoglobosins A and C, were detected in the synnemata. Forty of the SMs identified, including clavicipitic acid, expansolides A/B and C/D, roquefortines C and D, andrastins A, B, and C and eight communesins (A, B, D, E, F, I, K, and com470) were also detected in synnemata. Finally, unknown metabolites (14) with chemical formulae C_16_H_26_N_2_O_4_S_2_ (R_T_ = 22.02) C_18_H_16_N_2_O_2_ (R_T_ = 17.74), C_18_H_18_N_2_O_2_ (R_T_ = 14.61), C_19_H_38_O_6_ (R_T_ = 38.19), C_22_H_20_N_3_O (R_T_ = 38.56 and 39.29), C_23_H_24_N_2_O_6_ (R_T_ = 26.92), C_24_H_26_N_2_O_6_ (R_T_ = 33.79), C_26_H_40_O_6_ (R_T_ = 29.93), C_28_H_38_O_7_ (R_T_ = 35.32), C_28_H_38_O_8_ (R_T_ = 27.70, 28.44, and 29.57), C_29_H_31_N_5_O_6_ (R_T_ = 14.66) were found in the synnemata of the null mutant Pe∆*brlA* strain.

### 2.5. Analysis of the Transcriptome of PeΔbrlA

A microarray analysis was performed to evaluate the impact of *brlA* deletion on *P. expansum* transcriptome after five days of growth on MEA. The PeΔ*brlA* strain showed 918 up-expressed genes and 1398 down-regulated genes compared to WT strain. Genes were considered to be significantly differentially expressed when the Log_2_-fold change was < −1 or > 1 with a *p*-value < 0.05. Among these, 365 genes were regulated 10 or more times, with 322 genes down-regulated and 43 genes up-regulated, respectively. As the central genetic regulatory cascade BrlA → AbaA → WetA exists in *Aspergillus* species, we investigated the change in the expression of *abaA* and *wetA* in the null mutant strain. These genes were 12-fold and 14-fold down-regulated, respectively. As expected, a lot of down-regulated genes in the PeΔ*brlA* strain were related to conidiation (Table 4). 

Firstly, the deletion of *brlA* dramatically affected the expression of *rodA* and *rodB* genes that encode hydrophobins, the latter conferring a hydrophobic character to asexual spores. Except for the gene *abr2*, all the genes involved in 1,8-dihydroxynaphthalene (DHN)-melanin biosynthesis (*alb1*, *arp1*, *arp2*, *ayg1* and *abr1*) were strongly under-expressed compared to those in the WT strain. The velvet proteins (VeA, VelB, VelC, VosA) and their partner LaeA play a role in fungal development, more particularly in the balance between asexual and sexual reproduction in *A. nidulans* [60,61]. In *P. expansum*, only a slight decrease in the expression of the *vosA* gene and a slight increase in the expression of the *veA* gene occurred when the *brlA* gene was deleted. Trehalose is associated with conidiation, germination, and survival of asexual spores. Several studies have shown that WetA and VosA govern trehalose biosynthesis [62]. As the deletion of *brlA* affected the normal expression of *wetA* and *vosA* to a lesser extent, we focused on the expression of genes involved in trehalose biosynthesis such as *tpsA*, *orlA*, and *ccg-9* [62]. Only the expression of the latter was significantly reduced in the null mutant strain.

We also observed a decrease in the expression of *vadA*, a recently characterized spore-specific regulator [60], and significant up regulation of the VosA-repressed *dnjA* gene encoding the molecular chaperone [63].

Several examples of SMs specific to the spores have been reported in filamentous fungi [64,65]. Thus, we particularly focused on genes coding for backbone enzymes involved in secondary metabolism. In addition to the *alb1* gene mentioned above, the expression of nine backbone genes, *cnsF* (PEXP_030510), PEXP_018960, PEXP_095510, PEXP_095540, PEXP_072870, PEXP_006700, PEXP_037250, PEXP_029660, and PEXP_043150 was markedly altered in the PeΔ*brlA* strain. The DEGs were considered when the Log_2_-fold change was < −2 with a *p*-value < 0.05. One of these genes (*cnsF*) has been reported to be involved in communesin biosynthesis.

By contrast, six genes were significantly up-regulated (Log_2_-fold change was > 2 and a *p*-value < 0.05) when *brlA* was deleted, PEXP_074060, PEXP_096300, PEXP_045260, PEXP_028920, PEXP_063170, and PEXP_060620 (Table 5).

**Figure 6:**
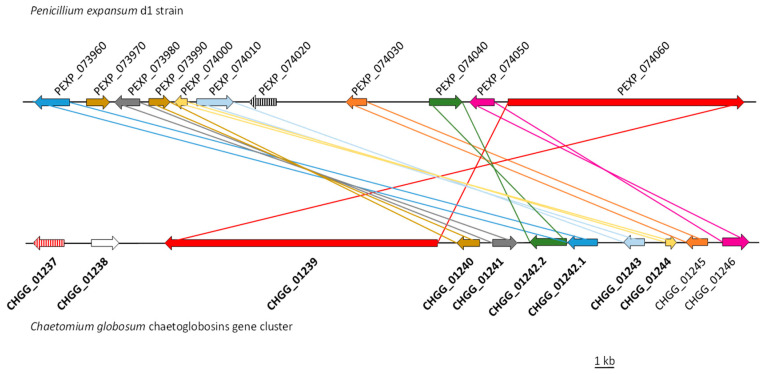
Comparison of the chaetoglobosin gene cluster in *Penicillium expansum* d1 strain and *Chaetomium globosum* strain. In bold, the cluster as described in Ishiuchi et al. [66].

AntiSMASH analysis showed that PEXP_074060 belongs to a putative biosynthetic gene cluster. This cluster is composed of 11 genes that were up-regulated in the null mutant compared to the WT strain (Table 6). To confirm the microarray results, the expression of all genes of the putative cluster were assessed by qPCR (Table S3, Figure S4). AntiSMASH analysis revealed also that this putative cluster shares features with the chaetoglobosin cluster [66]. A more detailed manual BlastP analysis showed that, except two genes, other chaetoglobosin genes have homologous genes located in the putative cluster (Figure 6).

BrlA was previously studied in two *Penicillium* subgenus *Penicillium* species: *P. digitatum* [38] and *Penicillium rubens* [49]. As transcriptome data from Δ*brlA* mutants were available for *P. digitatum* and *P. rubens*, data from the three species were compared. Without being exhaustive, Tables S4–S7 summarize this comparative study and are the subject of part of the discussion.

## 3. Discussion

Expressed for the earliest step of asexual reproduction in *Aspergillaceae* fungi, the *brlA* gene encodes a C_2_H_2_-type zinc-finger TF essential for conidiation. [37,68]. Although the *brlA* gene has been extensively studied, previous research focused on the *Aspergillus* genus ignored a wide field of research in other species. Here by creating a null mutant Pe∆*brlA* strain, we demonstrated that the *brlA* gene not only plays a fundamental role in the regulation of asexual development in *P. expansum*, but also influences the biosynthesis of certain SMs. The development of the null mutant Pe∆*brlA* strain on solid media results in a completely different phenotype from that of the WT strain. The *brlA* deletion resulted in a strain devoid of conidia, because in the absence of the *brlA* gene, the conidiogenesis process was stopped before metulae were created, which then formed only elongated aerial hyphae and gave the strain a “bristle-like” appearance. These results have also been reported in *A. niger* [69], *A. clavatus* [45], and *A. nidulans* [34] where inactivation of the *brlA* gene resulted in white aconidial strains resembling the Pe∆*brlA* strain in appearance. In genus *Penicillium*, the deletion of *brlA* in *P. digitatum* resulted also in a complete absence of conidiation [38]. Since the null mutant strain is entirely white, it can consequently be concluded that the *brlA* gene regulates the biosynthesis of conidia in *P. expansum*. In *P. decumbens*, the deletion of *brlA* produced strains that lack conidiophores [48]. During its development, the hyphae in the null mutant strain had more branches than the WT strain, but the hyphae were shorter displaying lower values of hypal growth length (Lhgu), evidence that removing *brlA* led to more frequent branching [48]. Preliminary studies in the *Aspergillus* genus have shown that *brlA* is an extremely important gene in the CRP of conidiation because it activates the expression of *abaA*, which in turn, activates *wetA*, the other two genes in this pathway, resulting in the reproduction and dissemination of the fungus [41,45]. The mutation of the regulation factors, AbaA and WetA, did not interfere in the formation of the vesicles, but eliminating *abaA* led to the formation of abnormal phialides that blocked the formation of conidia, while the mutation of *wetA* causes the spores to autolyze during the final stages of differentiation [34]. In *P. expansum*, *brlA* deletion led to a 12-fold and 14-fold decrease in the expression of the *abaA* and *wetA* genes, respectively.

As expected, the deletion of *brlA* also blocked the expression of other genes involved in conidiation. Among the genes most impacted by the deletion were genes encoding elements of the outer layer of conidia. This layer is composed of hydrophobins encoded by *rodA* and *rodB* and the DHN-melanin pigment is deposited beneath this hydrophobin layer. The induction of the DHN-melanin gene cluster by BrlA has already been identified in *A. fumigatus* [70] and in *P. decumbens* [48]. In *A. fumigatus*, the DHN-melanin pathway is encoded by six genes (*alb1*, *ayg1*, *arp2*, *arp1*, *abr1*, *abr2*) located in a cluster [71]. In *P. expansum*, five of them are scattered throughout the genome, with the exception of a cluster reduced to three or four genes depending on the strain studied. Therefore, there is no presence of an *abr2* orthologous gene in *P. expansum* suggesting that the DHN-melanin pathway stops before the last enzymatic step and leads to the synthesis of 1,8-DHN.

A weaker decrease in *axl2* gene expression was also observed in the null mutant strain. During *A. nidulans* conidiation, this transmembrane protein is localized to phialide-spore junctions. It is required for the septation event that splits the new conidia from their phialides. The *axl2* gene is over-expressed during conidiophore development in response to overexpression of *brlA* or *abaA* [72]. In *A. flavus*, *A. fumigatus* and *A. nidulans*, *vosA* is constantly induced by *wetA* [39]. AbaA is also required for expression of *vosA* [73]. Here, we observed a slight decrease in *vosA* gene expression in the null-mutant compared to the WT strain.

Efforts have been made for several years to identify target genes of VosA in the model species *A. nidulans*. This work led to the discovery of the proteins DnjA, VadA, and VidA. VosA activates *vadA* [60] and *vidA* [74] whereas it represses *dnjA* in *A. nidulans* [63]. In *P. expansum*, the deletion of *brlA* resulted in a lesser *vadA* and a higher *dnjA* transcripts level but did not affect *vidA* expression. The deletion of *brlA* also led to overexpression of the PEXP_047560 gene, a *zcfA* homolog. Discovered and characterized recently in *A. flavus* and *A. nidulans*, this Zn2Cys6 TF is essential for the balance between sexual and asexual reproduction. The authors showed that its level of transcripts increased in the Δ*vosA* mutant of each species [75].

While the role of BrlA is well documented in the genus *Aspergillus*, data on BrlA in the genus *Penicillium* are limited to three studies on *P. decumbens* [48], *P. rubens* (formerly identified as *P. chrysogenum*) [49], and *P. digitatum* [38]. Although together with *P. expansum*, *P. rubens*, and *P. digitatum* belong to the *Penicillium* subgenus *Penicillium*, *P. digitatum*, and *P. expansum* are phylogenetically closely related and are classified in the *Penicillium* section [76]. A recent study estimated that the two species diverged only about 15 million years ago (MYA) [77], while the *P. rubens* ancestor separated from the *P. digitatum* and *P. expansum* ancestors about 20 MYA. Following transcriptome analyses of the Δ*brlA* mutant of *P. rubens* [49] and *P. digitatum* [38], the lists of down- and up-regulated genes in the respective PeΔ*brlA* strains were compared. Of the 106 genes whose expression decreased with a Log_2_FC < −3 at PdΔ*brlA*, 101 have an orthologous gene in *P. expansum* and 79.2% of these were also significantly down-regulated (Log_2_FC < −1, adj. *p*-value < 0.05) in the mutant PeΔ*brlA* (Table S4). This suggests that the gene network under direct or indirect positive influence of BrlA factor has remained relatively unchanged for 15 million years. Surprisingly, if one considers now the genes under negative BrlA regulation, only 12 genes out of the 39 ortholog genes up-regulated in PdΔ*brlA* with a Log_2_FC > 2 showed an increase in their expression in PeΔ*brlA* (Table S5).

Sigl et al. [49] identified 38 genes regulated in a similar way to *wetA* in *P. rubens* Δ*brlA* (PrΔ*brlA*). Eighteen of these genes were also regulated in the same way in the PeΔ*brlA* strain (Table S6). Among them, we identified five genes (PEXP_018490, PEXP_030380, PEXP_096550, PEXP_096560, PEXP_037140) that have orthologous genes in *A. flavus*, *A. fumigatus*, and *A. nidulans*, and that were all under-expressed in the Δ*wetA* strain in all three species [39]. The role of these genes is not yet known. The only information available is that, except the PEXP_096560 gene, all orthologous genes were down-regulated at the conidiation stage in *A. fumigatus* Δ*atfA* strain [78]. In the same study, the authors also identified 93 genes regulated in a similar way to *abaA* in PrΔ*brlA*, of which 59 orthologous genes were down-regulated in PrΔ*brlA* and PeΔ*brlA* strains (Table S7). Only a few of these genes have been characterized to date. Among them, particular attention has been paid to *gin4* (PEXP_066390). This gene is also strongly down-regulated in PdΔ*brlA* (Log_2_FC = −4.9) and encodes a kinase conserved in Ascomycetes. It has been demonstrated that it phosphorylated septins in Saccharomycetales such as *Saccharomyces cerevisiae* [79]. In *A. fumigatus*, the deletion of *gin4* led to an increase in the interseptal distance [80]. Although a link between greater interseptal distance and hyphal radial growth has not been demonstrated, the lengthening of the interseptal distance could explain the higher radial growth observed in the PeΔ*brlA* strain in some media. The inability of PeΔ*brlA* strain to produce conidia led us to (i) the impossibility of generating a complemented mutant to restore the WT phenotype, (ii) the use of two types of inoculum with a risk to introduce a bias in the transcriptomic results. The strong similarities in the transcriptome data between the three mutants PeΔ*brlA*, PdΔ*brlA* [38], and PrΔ*brlA* [49] support the idea that the difference of inoculum had a minor impact and that it was indeed the deletion of *brlA* that caused the changes observed in the PeΔ*brlA* strain.

VeA is a global TF that is a member of the velvet complex involved in the regulation of many cellular processes, including SM biosynthesis and fungal development, which positively regulates sexual reproduction [61,81]. In *A. nidulans* VeA acts upstream of BrlA to inhibit asexual development. However, our results showed that BrlA had a negative effect on the expression of *veA* [60,61]. The slight increase in *veA* expression in the null mutant strain could explain the higher patulin production since patulin is strongly affected when the *veA* gene is deleted [26]. El Hajj Assaf et al. [26] have shown that, surprisingly, the null mutant Pe∆*veA* strain has lost the ability to create coremia, rigid structures formed by aggregation of conidiophores, both in vitro and in vivo. In the dark, the strain was still able to sporulate on synthetic media, but due to the absence of coremia, the null mutant strain was unable to pierce the epicarp of the apple and emerge from the fruit to complete its life cycle [26]. Our results showed that, although the absence of the *brlA* gene completely blocked the production of conidiophores, in vivo the mutant Pe∆*brlA* strain was able to produce rigid synnemata that allowed it to pierce the epicarp and emerge from the apple. To summarize the sequence of events that take place at the end of apple infection, VeA is essential for the formation of synnemata, which, in turn, is indispensable for perforation of the epicarp. BrlA is required in the second step to enable the formation of the entire fruiting structure, e.g., the conidiophore with all its components (rami, ramuli, metulae, phialides and conidia).

As *P. expansum* is the main cause of blue mold disease in apples and producer of patulin [16], we also analyzed the pathogenicity of the mutant Pe∆*brlA* strain in Golden Delicious apples. First, we observed that the null mutant strain was able to colonize the fruit, thereby inducing the disease, but differently from the WT strain. During the first six days, both strains showed the same development profile. From day nine on, a significant increase in the rot rate was observed in the null mutant strain, resulting in a final lesion diameter 20% larger than that of the WT strain. In addition, we found that the absence of the *brlA* gene in *P. expansum* did not reduce or stop patulin production. Pe∆*brlA* quadrupled compared to the WT strain. This observation is in agreement with reports that patulin is an important but not essential factor in the pathogenicity of *P. expansum* [21,22,82]. When the *patL* gene encoding the specific TF in the patulin biosynthesis pathway was deleted in *P. expansum*, patulin production was completely suppressed. The mutation also reduced virulence in apples inoculated with the null mutant strain. However, when patulin was added exogenously, the ability to cause disease was restored, suggesting that patulin plays a role in the development of apple spoilage [22]. These results were also observed in Golden Delicious apples infected with the null mutant strain PeΔ*veA*. Where the elimination of global FT VeA, involved in MS production, also suppressed patulin production and reduced the virulence of *P. expansum* [26]. Pathogenicity studies in 13 apple varieties showed that both the null mutant Pe∆*patL* and the WT strains were able to infect apples, but the intensity of symptoms depended not only on the capacity to produce patulin but also on the genetic background of the apple, suggesting that patulin is an aggressiveness factor rather than a virulence factor [22]. Several decades ago, conidiogenesis was linked to patulin production when a mutation at an early stage of conidiation caused a notable decrease in patulin production in *Penicillium griseofulvum* (syn = *P. urticae*) [83]. These results are in contradiction with the present results. Unfortunately, the mutant strain in the last study was generated by chemical mutagenesis and the mutation(s) has (have) not been genetically characterized for more in-depth discussion.

The stages of fruit ripening also influence the pathogenicity and virulence of the fungus, leading to increased accumulation of patulin in ripe fruits infected by *P. expansum* [25]. On the other hand, the availability of nutritional sources, such as carbon and nitrogen, is a key factor in the development of fungi and in the biosynthesis of SMs. When *P. griseofulvum* was grown on PDA and MEA media, conidiation and production of griseofulvin were reported to increase in media with higher carbon content (PDA) [84]. As apples are a good source of carbon, rich in glucose, sucrose, and fructose, we studied the growth profiles of the null mutant Pe∆*brlA* and WT strains in minimal media enriched with different carbon sources. The greatest development was observed in the APAM medium, perhaps because it is an apple-based natural medium. Media supplemented with glucose and fructose promoted the growth of the strains compared to the other monosaccharides. Surprisingly, when the strains were grown in a medium containing citrus pectin, their diameters were bigger than when they were grown in apple pectin. In 75% of the media tested, the null mutant strain developed significantly better than the WT strain. Other in vitro studies have also shown that high concentrations of sugars such as glucose and sucrose reduced the production and accumulation of SMs [23,25].

The production of natural metabolites in filamentous fungi is often linked to cell development and differentiation processes, as the environmental conditions required for sporulation and secondary metabolism are similar [28]. The most widely studied compounds are mycotoxins, due to their harmful effects on human and animal health. The relationship between sporulation and mycotoxin production has been assessed in several genera. The influence of several inhibitors of conidiophore maturation has been studied in *Aspergillus parasiticus*. At a concentration of 1 mg/mL of these inhibitors, both sporulation and aflatoxin B production were strongly affected, suggesting conidiogenesis and secondary metabolism are interrelated [85]. Recently, the suppression of early-acting regulators of sexual and asexual reproduction has been shown to be closely correlated with SM biosynthesis. For example, deletion of the *veA* gene in *A. niger* not only reduced ochratoxin A production and tolerance to oxidative stress, but also the production of conidia, as *brlA* gene expression was significantly reduced [86]. Satterlee et al. [87] reported that deletion of the *hbxA* gene that encodes a transcriptional developmental regulator, not only affected the biosynthesis of fumigaclavines, fumiquinazolins and chaetomine, but also reduced production of conidia since deletion resulted in under-expression of *brlA* and the fluffy genes *flbB*, *flbD*, and *fluG* in *A. fumigatus*. The Flb (For Fluffy low *brlA* expression) B and D are BrlA upstream development activators activated by FluG, which is responsible for the biosynthesis of an extracellular diffusible factor [88,89,90].

Our in vitro results showed that 50% of the SMs are produced by both the null mutant Pe∆*brlA* and the WT strains, meaning that the loss of *brlA* has no impact on the production of these compounds under the conditions tested here. The Pe∆*brlA* strain was unable to produce 32 compounds present in the WT strain, showing that BrlA is required for the production of these compounds. Conversely, BrlA negatively controlled the production of 32 other compounds only present in the null mutant strain. When the null mutant strain grew on dead vegetal biomass (wheat grains), the production of the mycotoxins patulin, citrinin, and roquefortines C and D as well as the bioactive compounds expansolides and andrastins A, B, and C were not inhibited by the suppression of the *brlA* gene. The compounds that were not produced by the null mutant strain were mainly communesins, of which only six of the 20 communesin derivatives produced by the WT strain were detected, in addition to the unknown metabolites of *m*/*z* 305.129 (R_T_ = 32.50 and 33.17) and 319.145 (R_T_ = 35.48 and 36.44), we suggest that these compounds may be related to pigmentation or spore protection. Among the new compounds not produced by the WT strain, we found a wide range of chaetoglobosins as well as compounds that could be members of the cytochalasan alkaloid family. The production of several chaetoglobosins has already been detected in different isolates of *P. expansum* [13] and these compounds have a wide range of biological activities, including antitumor, antifungal, or antibacterial properties [91].

The main compounds detected in the synnemata were chaetoglobosins, with 14 different derivatives (including chaetoglobosins A and C) whereas other derivatives are produced by the hyphae inside the apple. This may mean that biosynthesis of these metabolites takes place when the fungus emerges from the fruit. This observation suggests a spatial organization of this biosynthesis pathway. An example of selective accumulation of a particular secondary metabolite in a specific fungal tissue has already been reported [46]. Lim et al. [46] observed a predominant accumulation of fumiquinazoline C in the conidia of *A. fumigatus* whereas its biosynthetic precursors, fumiquinazolines A and F, were detected at comparable levels at different stages of development (basal hyphae, conidiophores, and conidia). Patulin and citrinin were detected only in apple flesh, not in synnemata. Disruption of the *brlA* gene in *A. fumigatus* not only yielded strains lacking conidiophores but that were also unable to produce the ergot alkaloids (festuclavine and fumigaclavines A, B, and C) fumiquinazoline C, trypacidin, and its two precursors (monomethylsulochrin questin), present in the conidia of the WT strain [64,92]. By contrast, strong production of fumitremorgins and verruculogen was reported [64]. The comparison of metabolome analyses of Pe∆*brlA* and WT cultures on sterilized labeled wheat grains evidenced the disappearance of some compounds and the appearance of other metabolites. Our results indicated that some SMs were specifically regulated by the *brlA* gene in *P. expansum* and confirmed that patulin production was not linked to the conidiogenesis. The in vivo analyses showed that its biosynthesis takes place in the vegetative mycelium inside fruits and stops when the competence phase begins.

A cluster of genes involved in the biosynthesis of chaetoglobosins has been identified in *P. expansum* [93]. It consists of seven genes (*cheA*-*cheG*). Surprisingly, only *cheF* (PEXP_043620) coding for a regulator is present in the eight *P. expansum* genomes sequenced and available in GenBank. Our transcriptomic analysis showed that this gene was very poorly expressed, and we observed no significant difference between the WT and the null mutant strains. These results were confirmed by qPCR. Additionally, several qPCR attempts using several primer designs were made to detect any expression of the other six *che* genes. All these attempts failed, suggesting that only *cheF* gene subsists in the genome of strain NRRL 35695. However, the production of chaetoglobosins by *P. expansum* is consistent, since another study showed that 100% of the strains originating from different substrates and geographical origins produced chaetoglobosins [13]. Instead, a cluster of 11 genes sharing high similarity with the gene cluster of chaetoglobosins in *Chaetomium globosum* [66] is present in *P. expansum* genomes. Although Ishiuchi et al. [66] delineated the cluster at nine genes, the two genes located directly downstream (CHGG_01245 and CHGG_01246) have a corresponding homolog in *P. expansum* (Figure 6). The difference between the clusters in the two species is the absence in *P. expansum* of a gene homologous to CHGG-01238 coding for a transposase and the absence of CHGG_01237 coding for a regulator. In *P. expansum*, the latter is replaced by another TF homologous to the cytochalasin cluster-specific regulator in *A. clavatus* [67]. The involvement of the transposase in chaetoglobosin biosynthesis has not been demonstrated to date. However, the absence of the gene CHGG-01238 in another chaetoglobosin-producing strain of *C. globosum* suggests that it is not essential for the synthesis of these compounds [94]. Our transcription analyses (microarray and qPCR) showed that all genes of this putative gene cluster were over-expressed in PeΔ*brlA*.

In this study, we also showed that the deletion of the *brlA* gene leads to over-production of chaetoglobosins with the appearance of minor compounds that were undetectable in the WT strain. Taken together, these data strongly suggest that this putative cluster is responsible for the biosynthesis of chaetoglobosins in *P. expansum.* To confirm this hypothesis and to determine the exact role of homologous proteins to CHGG_01245 and CHGG_01246, the generation of monogenic null mutants is currently underway.

## 4. Materials and Methods

### 4.1. Fungal Strains and Pe∆brlA Mutant Strain Construction

*Penicillium expansum* NRRL 35695, originally isolated from grape berries in Languedoc-Roussillon (France) was used as a wild type strain (WT). To understand and study the role of the *brlA* gene in *P. expansum*, a gene deletion strategy was applied in the WT *P. expansum* NRRL 35695 strain. Considering that BrlA is conserved in *Aspergillaceae* [50], the sequence for *P. expansum brlA* (PEXP_049260) were obtained from the genomic sequence of *P. expansum* strain d1 [21] after a BlastP analysis using the previously characterized *A. fumigatus* (AFU1G16590) [95] and *P. rubens* (PC06g00470) [49] BrlA proteins. The protein encoded by PEXP_ 049260 shares 95.5% identity with BrlA (PC06g00470) from *P. rubens* and 60% identity with BrlA (AFU1G16590) from *A. fumigatus*, respectively.

The construction of the null mutant strain is detailed in Supplementary Materials (Figure S5). Briefly, using the homologous recombination strategy, the *brlA* gene was replaced by the hygromycin resistance marker (*hph*), flanked by the DNA sequences corresponding to the 5′ upstream and 3′ downstream sequences of the *brlA* coding sequence. The gene disruption cassette was constructed by PCR, whereby the flanking regions 5′ upstream and 3′ downstream were amplified from the genomic DNA of *P. expansum* strain NRRL 35695. The pAN7.1 plasmid was used to generate the amplicon containing the hygromycin resistance gene [96], subsequently all fragments were assembled using double-joint PCR [97,98]. The cassette in which the *brlA* gene was replaced by the hygromycin resistance marker was used to transform the WT strain according to the method described by Snini et al. [22].

Contrary to usual practice, a complemented strain was not generated. To generate a complemented mutant, we have to transform protoplasts prepared 12 h after inoculation of conidia, but the null mutant PeΔ*brlA* was no longer able to produce the conidia essential for the formation of protoplasts.

### 4.2. Validation of Pe∆brlA Mutant Strain

In order to confirm the insertion of the hygromycin marker at the *brlA* locus of *P. expansum* and the deletion of the *brlA* gene, only the transformants that exhibited morphological characteristics different from those of the WT, e.g., the strains were white, with a “bristle-like” appearance and devoid of conidiophores, were molecularly or genetically tested. Figure S6 details the results obtained by PCR screening in the WT and Pe∆*brlA* strains. PCR with primers specific to the *brlA* gene, dBrlA-geneF/dBrlA-geneR (Table S8), generated a 1137 base pair (bp) fragment for the WT strain, while no fragment for the null mutant Pe∆*brlA* strain was generated. Amplification of 5′ and 3′ locus *brlA*/*hph* junctions in Pe∆*brlA* strain displayed fragments of 2121 bp and 2185 bp, confirming the replacement of *brlA* by *hph*.

Validation of the transformants by genome walking confirmed the correct insertion of the selection marker at the *brlA* locus, the EcoRV library generated an amplicon of 623 bp, while the PvuII library generated an amplicon of 4009 bp (Figure S7). The restriction cutting for the EcoRV library produced fragments of 217 and 483 bp with the enzyme KpnI and of 276 and 424 bp with the enzyme BstxI. For the PvuII library, the enzyme HindIII produced fragments of 298 and 3702 bp and the enzyme BamHI of 929, 1159, and 2004 bp (Figure S7). These results show that there is only one copy of the disruption cassette in *P. expansum* and that it is integrated at the locus *brlA*.

The final validation of the transformants was performed by qPCR analysis. Figure S8 confirms the absence of *brlA* gene expression in the null mutant Pe∆*brlA* strain. This observation was confirmed in the microarray analysis (Log_2_FC = −6.21; adjusted *p*-value 1.07× 10^−14^).

### 4.3. Macroscopic and Microscopic Morphology

The WT and Pe∆*brlA* strains were grown in MEA (Biokar diagnostics, Allonne, France; 30 g/L malt extract, 15 g/L agar) Petri dishes for seven days at 25 °C, after which spore suspension was made of the WT strain and its concentration was quantified using a Malassez cell [99]. MEA, PDA (Merck KGaA, Darmstadt, Germany; 30 g/L potato extract, 15 g/L agar), and CYA [10 mL/L concentrated Czapek (30 g/L NaNO_3_, 5 g/L MgSO_4_ 7H_2_O, 0.1 g/L FeSO_4_, 5 g/L KCl), 1 mg/L K_2_HPO_4_, 5 g/L yeast extract, 30 g/L saccharose, 15 g/L agar] media were inoculated centrally with 10 µL of a 10^6^ spores/mL suspension of the WT strain or 5 mm^2^ of mycelium from the mutant Pe∆*brlA* strain and were incubated for 10 days at 25 °C in the dark. Microscopic characteristics were observed using an optical microscope CX41 (×400 and ×1000) (Olympus, Rungis, France) after seven days. Macroscopic characteristics were studied using a stereomicroscope SZX9 (×12–120) (Olympus), after 10 days. The experiment was performed in triplicate.

### 4.4. Pathogenicity Study and Patulin Production

An in vivo study was performed to investigate the impact of the *brlA* gene mutation on the aggressiveness of the blue mold caused by *P. expansum*. Golden Delicious apples were purchased in a supermarket (Carrefour, Toulouse, France) and wash-sterilized in a 2% sodium hypochlorite solution [82]. To obtain equivalent study conditions, first, a few spores of the WT strain or a fragment of mycelium of the mutant Pe∆*brlA* strain were placed in 50 mL of a liquid yeast extract glucose medium (Merck KGaA; 5 g/L yeast extract, 20 g/L glucose) on an orbital shaker set at 150 rpm at 25 °C for 72 h. Then, 50 mg of mycelium of each strain was weighed and placed in 3 mL of 0.05‰ Tween 80 and sonicated for 5 h in an ultrasonic sonicator (Bransonic 221 Ultrasonic bath, Roucaire, Les Ulis, France). Apples were wounded with a sterile toothpick on one side and 10 µL of the suspension was deposited. Infected apples were incubated for 14 days at 25 °C in the dark. The diameter of the rotten spots was measured daily and the volume of rot was determined at the end of the incubation period [16]. At the end of the incubation period, the whole apples were ground in a blender into puree, and an aliquot (10 g) of each sample was analyzed for patulin production as described by Snini et al. [22] and El Hajj Assaf et al. [26]. Patulin was quantified by HPLC as described previously [22,26]. The experiment was performed with nine biological replicates of each strain.

### 4.5. Analysis of Growth on Different Carbon Sources

The WT and null mutant Pe∆*brlA* strains were grown in Petri dishes containing minimal medium (MM) [6.0 g/L NaNO_3_, 1.5 g/L KH_2_PO_4_, 0.5 g/L KCl, 0.5 g/L MgSO_4_, 200 µL/L trace elements (10 g/L EDTA, 4.4 g/L ZnSO_4_·7H_2_O, 1.01 g/L MnCl_2_·4H_2_O, 0.32 g/L CoCl_2_·6H_2_O, 0.315 g/L CuSO_4_·5H_2_O, 0.22 g/L (NH_4_)_6_Mo_7_O_24_·4H_2_O, 1.47 g/L CaCl_2_·2H_2_O, 1.0 g/L FeSO_4_·7H_2_O), 10 g/L glucose, 15 g/L agar] [100] supplemented with different carbon sources. Glucose, galactose, fructose, rhamnose, and xylose were used as monosaccharides at a final concentration of 25 mM. The polysaccharides: cellulose, starch, citrus pectin, apple pectin, and LBG were added at a final concentration of 0.5% [101]. The APAM medium, an apple-based substrate permissive for patulin production, was prepared as described by Baert et al. [16]. The media were inoculated centrally with 10 µL of a 10^6^ spores/mL suspension of the WT strain or 5 mm^2^ of mycelium from the mutant Pe∆*brlA* strain and incubated at 25 °C for seven days in the dark. The diameters of the colonies were measured at the end of the incubation period. All the experiments were conducted in triplicate.

### 4.6. Secondary Metabolism Study

#### 4.6.1. Fungal Growth Conditions on Labeled Wheats

The SMs were analyzed using a non-targeted metabolomic approach combining LC-HRMS. Known and unknown metabolites produced by the fungus were detected and unambiguously characterized by a unique chemical formula. Briefly, wheat grains labeled with stable isotopes were used, 96.8% ^13^C enriched wheat (^13^C wheat) and 53.4% ^13^C and 96.8% ^15^N wheat (^13^C/^15^N wheat); 99% ^12^C (^12^C wheat) natural wheat grains were also used. The natural and labeled wheat grains were produced and treated as described previously [102,103]. Ten grams aliquots of each type of sterilized wheat grains were placed in sterile Petri dishes (45 mm diameter) and inoculated 100 µL of a 10^5^ spores/mL suspension of the WT strain or three fragments (5 mm^2^) of mycelium from the mutant Pe∆*brlA* strain. The cultures were incubated at 25 °C for 14 days in the dark; a Petri dish containing uninfected ^12^C natural wheat grains was used as control. After 14 days, the substrate is fully colonized by each strain, reducing the bias introduced by the use of two types of inoculum. At the end of the incubation period, fungal metabolites were extracted according to the methodology described previously [103].

#### 4.6.2. In Vivo Production of Secondary Metabolites

The detection of SMs in vivo was performed on Golden Delicious apples. The fruits were treated as detailed above and wounded with a sterile toothpick. The apples were inoculated with 20 µL of a suspension (as detailed in Section 4.4.) from the null mutant PeΔ*brlA* strain. The infected apples were incubated for 30 days at 25 °C in the dark. At the end of incubation, the synnemata (Pe∆*brlA*) were collected on nylon membrane (UptiDisc nylon membrane, 47 mm diameter; Interchim, Montluçon, France) using a vacuum pump and extracted with 50 mL ethyl acetate for 72 h. Apples were ground [104] and SMs were extracted as detailed in Snini et al. [22]. Four biological replicates were performed.

#### 4.6.3. Analytical Parameters for LC-HRMS

The extracts were analyzed by LC-HRMS. The chromatographic system consisted in an ultimate 3000 HPLC device (Dionex/Thermo Scientific, Courtaboeuf, France). A gradient program of water acidified with 0.05% formic acid (phase A) and acetonitrile acidified with 0.05% formic acid (phase B) was used at 30 °C with a flow rate of 0.2 mL/min as follows: 0 min 20% B, 30 min 50% B, 35 min 90% B, from 35 to 45 min 90% B, 50 min 20% B, from 50 to 60 min 20% B. A 10 µL aliquot of each sample diluted twice with the mobile phase A was injected into a reversed-phase Luna^®^ C18 column (125 × 2 mm × 5 µm) (Dionex/Thermo Scientific). The mass spectrometer corresponded to an LTQ Orbitrap XL (Dionex/Thermo Scientific) fitted with an Electrospray Ionization Source (ESI) in the positive and negative modes. For the negative mode, the ionization parameters were set as follows: spray voltage: 3.7 kV, capillary temperature: 350 °C, sheath gas flow rate (N_2_): 30 arbitrary units (a.u.), auxiliary gas flow rate: 10 a.u. (N_2_), capillary voltage: −34 V and tube lens offset: −180 V. For the positive mode, the ESI parameters were set as follows: spray voltage: 4 kV, capillary temperature: 300 °C, sheath gas flow rate (N_2_): 55 arbitrary units (a.u.), auxiliary gas flow rate: 10 a.u. (N_2_), capillary voltage: 25 V and tube lens offset: −100 V. High resolution mass spectra were acquired between *m*/*z* 100 and 800 at a resolution of 7500. MS/MS spectra were obtained with the collision induced dissociation (CID) mode of the ion trap analyzer at low resolution and a normalized collision energy of 35%. The mass spectrometer was calibrated using the Thermo Fisher Scientific protocol.

#### 4.6.4. Parameters for High Performance Liquid Chromatography-Diode Array Detector (HPLC-DAD)

For patulin detection, the chromatography apparatus Ultimate 3000 HPLC system (Dionex/Thermo Scientific) equipped with a detector DAD was used. The presence of patulin was monitored at a wavelength of 277 nm with a 250 mm × 4.60 mm Gemini^®^ 5 µm C6-Phenyl column (Phenomenex, Torrance, CA, USA) at a flow rate of 0.9 mL/min at 30 °C. Eluent A was water acidified with 0.2% acetic acid and eluent B was HPLC-grade methanol (Thermo Fisher Scientific). The elution conditions were as follows: 0 min 0% B, 8 min 0% B, 20 min 15% B, 25 min 15% B, 35 min 90% B, from 35 to 40 min 90% B, from 45 to 60 min 0% B. The presence of patulin was confirmed by its retention time (min) and UV spectrum according to an authentic standard (Merck KGaA). Patulin concentration was calculated based on a standard curve.

#### 4.6.5. Identification of Fungal Metabolites

Results obtained from ^12^C, ^13^C and ^13^C/^15^N cultures were compared using our in-house MassCompare program to determine the elemental composition of each compound with a mass measurement accuracy of 5 ppm [103,105]. The metabolites were identified based on the MS/MS spectrum, chemical formulae, retention times, the MS/MS fragmentation pattern of the standard compound, AntiBase 2012 database [106], and the literature.

### 4.7. Identification of Secondary Metabolites Clusters

In order to identify the different SM clusters, an antiSMASH (Antibiotics-Secondary Metabolites Analysis Shell) analysis [107] was performed on *P. expansum* d1 genome [21].

### 4.8. Microarray Gene Expression Studies

The WT and null mutant Pe∆*brlA* strains were pre-cultured on MEA medium for seven days at 25 °C in the dark, after which a spore suspension was made with the WT strain. The mycelium of the null mutant strain was used as inoculation material as this strain does not produce conidia. Petri dishes containing MEA covered with sterile cellophane sheets were inoculated with 10 µL of a 10^6^ spores/mL suspension of the WT strain or 5 mm^2^ mycelium of the null mutant strain. The strains were incubated at 25 °C for five days in the dark. Total RNA was isolated at the end of the growth period. The mycelium was transferred to lysing matrix D tubes (1.4 mm ceramic spheres, Thermo Fisher Scientific), to which 760 µL of lysis buffer [10 μL of β-mercaptoethanol (Applied Biosystem, Thermo Fisher Scientific) and 750 μL of RLT buffer (Rneasy mini kit, QIAGEN, Courtaboeuf, France)] were added, and the tubes were placed in liquid nitrogen. The mycelium cells were homogenized in a Precellys homogenizer (Bertin Technologies, Montigny-le-Bretonneux, France) with three grindings at a speed of 6500 rpm for 15 s followed by 5 min incubation on ice, at 6500 rpm for 25 s and 5 min on ice, and a final 6500 rpm for 15 s. The samples were subsequently centrifuged at 16,000× *g* at 4 °C for 10 min. The supernatant was recovered in QIAshredder spin columns (QIAGEN) and the total RNA was purified using RNeasy spin minicolumns (QIAGEN) as described by Tannous et al. [20]. RNA quality was checked by electrophoresis using 4200 TapeStation System (Agilent Technologies, Les Ulis, France) and the concentration was determined using a Dropsense™ 96 UV/VIS droplet reader (Trinean, Ghent, Belgium).

Gene expression profiles were performed at the GeT-TRiX facility (GénoToul, Génopole Toulouse Midi-Pyrénées, France). Briefly, each sample was prepared from 200 ng of total RNA following procedure previously described by Tannous et al. [108] using Agilent Technologies instructions and hybridized on Agilent Sureprint G3 Custom microarrays (8 × 60 K, design 085497) of 62,976 spots following the manufacturer’s instructions. The expression analysis was performed with five and six biological replicates for PeΔ*brlA* and WT strains, respectively.

Microarray data and experimental details are available in NCBI’s Gene Expression Omnibus [109] and are accessible through Gene Expression Omnibus (GEO) Series accession number GSE155057 (https://www.ncbi.nlm.nih.gov/geo/query/acc.cgi?acc=GSE155057). The list of DEGs is available as supplementary material (Table S9).

Considering that transcriptome data were available for PdΔ*brlA* [38] and PrΔ*brlA* [49] mutants, the microarray results were compared to those obtained in these previous studies. For *P. digitatum*, the differentially expressed genes in PdΔ*brlA* (with Log_2_FC < −3 and > 2) were extracted from Table S2 linked to Wang et al. publication [38] and a search of *P. expansum* homologous genes was carried out by BlastP. The *P. expansum* genes homologous to genes significantly down-regulated in PdΔ*brlA* were listed in Table S4 and those homologous to genes significantly up-regulated in PdΔ*brlA* were listed in Table S5.

As the CRP BrlA → AbaA → WetA exists in *Penicillium* species, we compared our results with genes identified as similarly regulated to *abaA* (Table S6 from [49] and with those similarly regulated to *wetA* (Table S5 from [49]) in PrΔ*brlA*. The *P. expansum* homologous genes to genes similarly regulated to *wetA* and *abaA* in PrΔ*brlA* were listed in Tables S6 and S7, respectively.

### 4.9. Statistical Analysis of Microarray Data

Microarray data were analyzed using R and Bioconductor packages [110] as described in GEO accession GSE155057. Raw data (median signal intensity) were filtered, log2 transformed, and normalized using smooth quantile normalization (qsmooth) method [111]. A model was fitted using the limma lmFit function [112]. Pair-wise comparison between biological conditions was applied using specific contrast. A correction for multiple testing was applied using the Benjamini-Hochberg (BH) procedure [113] to control for False Discovery Rate (FDR). Probes with FDR ≤ 0.05 were considered to be differentially expressed between conditions.

### 4.10. Statistical Analysis

A Student’s test and one-way analysis of variance (ANOVA) were used to analyze the differences between the WT and the null mutant Pe∆*brlA* strains. Differences were considered to be statistically significant with a *p*-value ≤ 0.05. Statistical analysis of the data was performed using GraphPad Prism 4 software (GraphPad Software, La Jolla, CA, USA).

## 5. Conclusions

In conclusion, we showed in this study that *brlA* suppression led to no development of conidiophores but had no impact on synnemata formation. This effect has previously been described in other *Aspergillus* and *Penicillium* species. Transcriptome analysis showed that the gene network under the positive influence of BrlA was relatively conserved in *Penicillium* subgenus *Penicillium* and that many of them were genes involved in conidiation such as *wetA*, *abaA*, hydrophobin encoding genes, and melanin-like pigments encoding genes.

Metabolome and transcriptome analyses showed that *brlA* suppression resulted in altered communesin biosynthesis counterbalanced by enhanced chaetoglobosin production, unveiling a putative chaetoglobosin gene cluster. This study demonstrated that patulin production was not affected by inhibition of conidiation, confirming that there is no link between the biosynthesis of this mycotoxin and conidiogenesis. Furthermore, the absence of patulin in synnemata suggests that patulin was produced by hyphae when the fungus grew in the flesh of the apple and that its production stopped when the fungus was released from the fruit as synnemata.

## Figures and Tables

**Table 1:** Comparison of secondary metabolites detected in WT NRRL35695 and Pe∆*brlA* strains after culture on labeled wheat grains.

Molecular Formula	^12^C *m*/*z* (Da)	^a^ R_T_ (min)	Proposed Identification	WT	Pe∆*brlA*	Molecular Formula	^12^C *m*/*z* (Da)	^a^ R_T_ (min)	Proposed Identification	WT	Pe∆*brlA*
**C_7_H_6_O_4_**	**153.01919**	**3.59**	**Patulin ^†^**	+	+	C_28_H_32_N_4_O	441.26589	17.14	Communesin F ^d^	+	ND
C_7_H_8_O	109.06509	7.11	m-Cresol ^†^	+	+	C_28_H_32_N_4_O_2_	457.26116	23.65	Communesin A ^f,†^	+	+
C_7_H_8_O_2_	125.05998	6.79	m-Hydroxybenzyl alcohol ^†^	+	+	C_28_H_32_N_4_O_3_	473.25566	15.50		+	ND
C_7_H_8_O_3_	141.05493	3.80	Gentisyl alcohol ^†^	+	+	C_28_H_36_N_4_O_4_	493.27971	31.47	Fungisporin A or cyclo(VFVF)	+	+
C_7_H_8_O_4_	157.04990	2.67	Ascladiol ^†^	+	+	C_28_H_38_N_4_O_5_	511.29099	18.01	VAL-PHE-VAL-PHE	+	+
C_7_H_10_O_3_	143.07061	4.52		+	+	C_28_H_38_N_4_O_6_	527.28755	12.46	VAL-PHE-VAL-TYR	+	+
C_10_H_17_NO_5_	232.11872	8.83		+	+	**C_28_H_38_O_7_**	**485.25405**	**35.32**		+	+
C_10_H_17_NO_5_	232.11872	9.65		+	ND	C_28_H_38_O_7_	487.27063	36.21	Andrastin A ^†^	+	+
C_13_H_14_O_5_	251.09108	22.13	Citrinin ^†^	+	+	**C_28_H_38_O_8_**	**501.24792**	**27.70**		+	+
C_15_H_18_N_2_	227.15514	6.44	Aurantioclavine ^†^	+	ND	**C_28_H_38_O_8_**	**501.24792**	**28.44**		+	ND
C_15_H_19_NO_6_	310.12939	12.78		+	+	**C_28_H_38_O_8_**	**501.24786**	**29.57**		+	+
C_15_H_19_NO_6_	310.12964	14.80		+	+	**C_28_H_40_O_6_**	**471.27434**	**39.48**	**Andrastin C**	+	+
C_15_H_20_O_4_	265.14412	15.91	Expansolide C/D	+	+	**C_28_H_40_O_7_**	**487.26898**	**30.58**	**Andrastin B**	+	+
C_15_H_20_O_4_	265.14410	16.91		+	ND	C_29_H_27_N_5_O_5_	526.20693	15.70		ND	+
C_15_H_20_O_4_	265.14415	18.49	Expansolide C/D	+	+	C_29_H_27_N_5_O_5_	526.20689	17.61		ND	+
C_15_H_20_O_4_	265.14414	19.35		+	+	C_29_H_31_N_5_O_5_	530.23837	14.83		ND	+
C_16_H_18_N_2_O_2_	271.14496	7.62	Clavicipitic acid ^†^	+	+	C_29_H_31_N_5_O_5_	530.23812	15.85		ND	+
C_16_H_18_N_2_O_2_	271.14467	8.45	Clavicipitic acid ^†^	+	+	C_29_H_31_N_5_O_5_	530.23840	16.68		ND	+
C_16_H_26_N_2_O_4_S_2_	375.14202	22.02		+	+	C_29_H_33_N_5_O_6_	548.24883	11.36		ND	+
C_17_H_22_O_5_	307.15471	27.39	Expansolide A/B	+	+	C_29_H_33_N_5_O_6_	548.24860	12.14		ND	+
C_17_H_22_O_5_	307.15504	30.19	Expansolide A/B	+	+	C_29_H_33_N_5_O_6_	548.25180	15.42		+	ND
C_17_H_23_N_3_O_3_	318.17943	3.12		ND	+	C_31_H_36_N_4_O_2_	497.29061	31.66	Putative new undetermined communesin	+	ND
C_17_H_23_N_3_O_3_	318.18043	25.05		ND	+	C_32_H_34_N_4_O_3_	523.27152	31.84	Communesin D ^g^	+	+
C_18_H_16_N_2_O_2_	293.12911	13.58		+	ND	C_32_H_36_N_2_O_4_	513.27635	39.10	Chaetoglobosin J or Prochaetoglobosin III	+	+
C_18_H_16_N_2_O_2_	293.12915	17.74		+	ND	C_32_H_36_N_2_O_5_	529.26776	20.92	Chaetoglobosin B/G	+	+
C_18_H_16_N_2_O_3_	309.12421	14.76		+	ND	C_32_H_36_N_2_O_5_	529.26807	23.19	Chaetoglobosin B/G	ND	+
C_18_H_18_N_2_O_2_	295.14479	14.61		+	+	C_32_H_36_N_2_O_5_	529.27058	25.21	Chaetoglobosin B/G	+	+
C_18_H_31_NO_7_	374.21663	23.72		+	+	C_32_H_36_N_2_O_5_	529.27029	26.71	Chaetoglobosin B/G	+	+
C_18_H_35_N_3_O_4_	358.26901	3.54		ND	+	C_32_H_36_N_2_O_5_	529.26757	29.56	Chaetoglobosin B/G	+	+
C_19_H_16_N_2_O_2_	305.12911	32.50		+	ND	C_32_H_36_N_2_O_5_	529.27077	30.51	Chaetoglobosin B/G	+	+
C_19_H_16_N_2_O_2_	305.12933	33.17		+	ND	C_32_H_36_N_2_O_5_	529.26807	32.43	Chaetoglobosin B/G	ND	+
C_19_H_16_N_2_O_4_	337.11909	13.73		+	ND	C_32_H_36_N_2_O_5_	529.27067	33.36	Chaetoglobosin A ^†^	+	+
C_19_H_16_N_2_O_4_	337.11916	15.20		+	ND	C_32_H_36_N_2_O_5_	529.26771	34.25	Chaetoglobosin B/G	ND	+
C_19_H_20_O_5_	329.13738	33.29		ND	+	C_32_H_36_N_2_O_5_	529.26769	35.49	Chaetoglobosin B/G	ND	+
C_19_H_21_NO_7_	376.13901	17.45		+	+	C_32_H_36_N_2_O_5_	529.27063	36.41	Chaetoglobosin C ^†^	+	+
C_19_H_21_NO_7_	376.13904	18.65		+	+	C_32_H_36_N_2_O_5_	529.27056	37.41	Chaetoglobosin B/G	+	+
**C_19_H_38_O_6_**	**361.25871**	**37.23**		+	ND	C_32_H_36_N_2_O_5_	529.26814	38.29	Chaetoglobosin B/G	ND	+
**C_19_H_38_O_6_**	**361.25819**	**38.19**		+	ND	C_32_H_36_N_2_O_6_	545.26332	27.60	Putative cytochalasan	+	+
C_20_H_18_N_2_O_2_	319.14588	35.48		+	ND	C_32_H_36_N_2_O_6_	545.26241	29.23	Putative cytochalasan	ND	+
C_20_H_18_N_2_O_2_	319.14502	36.44		+	ND	C_32_H_36_N_2_O_6_	545.26288	30.57	Putative cytochalasan	+	+
C_20_H_21_NO_9_	420.12857	19.29		+	+	C_32_H_36_N_2_O_6_	545.26281	31.50	Putative cytochalasan	+	+
C_20_H_21_NO_9_	420.12858	22.19		ND	+	C_32_H_36_N_2_O_6_	545.26294	32.48	Putative cytochalasan	ND	+
C_20_H_26_O_8_	395.17142	13.61		+	+	**C_32_H_36_N_2_O_6_**	**543.24836**	**34.05**	Putative cytochalasan	+	+
C_22_H_23_N_5_O_2_	390.19390	15.09	Roquefortine C ^†^	+	+	C_32_H_36_N_4_O_2_	509.29257	34.95	Communesin B ^g,†^	+	+
C_22_H_25_N_5_O_2_	392.20913	9.99	Roquefortine D	+	+	C_32_H_38_N_2_O_4_	515.28895	35.59	Putative cytochalasan	+	+
C_23_H_24_N_2_O_6_	425.17179	26.92		+	+	C_32_H_38_N_2_O_4_	515.28890	37.93	Putative cytochalasan	+	+
**C_24_H_26_N_2_O_6_**	**437.17091**	**33.79**		+	ND	C_32_H_38_N_2_O_4_	515.28909	38.15	Putative cytochalasan	+	+
C_26_H_30_N_4_	399.25568	19.94	Communesin K ^b^	+	ND	C_32_H_38_N_2_O_5_	531.28384	21.75	Putative cytochalasan	ND	+
C_26_H_30_N_4_O	415.25034	14.62	Communesin I ^c^	+	+	C_32_H_38_N_2_O_5_	531.28990	22.76	Putative cytochalasan	+	+
C_26_H_30_N_4_O	415.25033	18.43	Communesin I ^c^	+	+	C_32_H_38_N_2_O_5_	531.28431	23.91	Putative cytochalasan	ND	+
C_26_H_32_O_8_	473.21511	9.67		ND	+	C_32_H_38_N_2_O_5_	531.28360	27.38	Putative cytochalasan	+	+
C_26_H_32_O_8_	473.21552	10.64		ND	+	C_32_H_38_N_2_O_5_	531.28354	28.02	Chaetoglobosin E ^h^	ND	+
C_26_H_32_O_8_	473.21489	34.14		ND	+	C_32_H_38_N_2_O_5_	531.28340	28.52	Putative cytochalasan	+	+
C_26_H_40_O_6_	449.28939	29.93		+	+	C_32_H_38_N_2_O_5_	531.28338	31.56	Penochalasin F ^h^	ND	+
C_27_H_29_N_5_O_4_	488.22783	10.80		ND	+	C_32_H_38_N_2_O_6_	547.27864	23.19	Putative cytochalasan	ND	+
C_27_H_29_N_5_O_4_	488.22766	11.03		ND	+	C_32_H_38_N_2_O_6_	547.27841	25.47	Putative cytochalasan	ND	+
C_27_H_30_N_4_O_2_	443.24564	15.03	Communesin E ^d^	+	+	C_32_H_38_N_2_O_6_	547.27842	27.09	Putative cytochalasan	ND	+
C_28_H_30_N_4_O_3_	471.23997	19.56	Com470 ^e^	+	+	C_32_H_38_N_2_O_6_	547.27851	28.27	Putative cytochalasan	ND	+
C_28_H_31_N_5_O_5_	518.24108	16.36		+	+	C_33_H_38_N_4_O_5_	571.29324	18.22	Com570 ^e^	+	ND
C_28_H_31_N_5_O_5_	518.24092	17.21		+	+	C_37_H_42_N_4_O_5_	623.32511	29.52	Com622 ^e^	+	ND

Compounds detected by negative electrospray ionization (ESI-) are in **bold**. **^a^** R_T_ = retention time, **^b^** Communesin K [53], **^c^** Communesin I [53,54], **^d^** Communesin E, and Communesin F [55], **^e^** Com470, Com570 and Com622 [56], **^f^** Communesin A [57], **^g^** Communesin D, and Communesin B [58]. **^h^** Chaetoglobosin E and Penochalasin F [59]. ^†^ Identified by standard. + = Detected. ND = Not detected.

**Table 2:** Secondary metabolites detected in Golden Delicious apples infected with the null mutant Pe∆*brlA* strain (30 dpi).

Molecular Formula	^12^C *m*/*z* (Da)	R_T_ (min) ^a^	Proposed Identification	Molecular Formula	^12^C *m*/*z* (Da)	R_T_ (min) ^a^	Proposed Identification
**C_7_H_6_O_4_**	**153.01919**	**3.59**	**Patulin**	C_29_H_27_N_5_O_5_	526.20689	17.61	
C_7_H_8_O_3_	141.05493	3.80	Gentisyl alcohol	C_32_H_36_N_2_O_4_	513.27635	39.10	Chaetoglobosin J or Prochaetoglobosin III
C_7_H_8_O_4_	157.04990	2.67	Ascladiol	C_32_H_36_N_2_O_5_	529.26776	20.92	Chaetoglobosin B/G
C_10_H_17_NO_5_	232.11872	8.83		C_32_H_36_N_2_O_5_	529.26807	23.19	Chaetoglobosin B/G
C_13_H_14_O_5_	251.09108	21.70	Citrinin	C_32_H_36_N_2_O_5_	529.27029	26.71	Chaetoglobosin B/G
C_16_H_26_N_2_O_4_S_2_	375.14202	22.02		C_32_H_36_N_2_O_5_	529.26898	29.54	Chaetoglobosin B/G
C_17_H_22_O_5_	307.15471	27.39	Expansolide A/B	C_32_H_36_N_2_O_5_	529.27539	30.13	Chaetoglobosin B/G
C_17_H_22_O_5_	307.15504	30.19	Expansolide A/B	C_32_H_36_N_2_O_5_	529.26807	32.43	Chaetoglobosin B/G
C_19_H_21_NO_7_	376.13901	17.45		C_32_H_36_N_2_O_5_	529.26769	35.49	Chaetoglobosin B/G
C_19_H_21_NO_7_	376.13904	18.65		C_32_H_36_N_2_O_5_	529.27056	37.41	Chaetoglobosin B/G
C_22_H_23_N_5_O_2_	390.19390	15.09	Roquefortine C	C_32_H_36_N_4_O_2_	509.29257	36.01	Communesin B
C_23_H_24_N_2_O_6_	425.17179	26.92		C_32_H_38_N_2_O_5_	531.28384	21.75	Putative cytochalasan
**C_28_H_38_O_7_**	**485.25405**	**35.32**		C_32_H_38_N_2_O_5_	531.28431	23.91	Putative cytochalasan
C_28_H_38_O_7_	487.27063	36.21	Andrastin A	C_32_H_38_N_2_O_6_	547.27864	23.19	Putative cytochalasan
**C_28_H_38_O_8_**	**501.24792**	**28.44**		C_32_H_38_N_2_O_6_	547.27841	25.47	Putative cytochalasan
**C_28_H_38_O_8_**	**501.24786**	**29.57**		C_32_H_38_N_2_O_6_	547.27842	27.09	Putative cytochalasan
**C_28_H_40_O_7_**	**487.26898**	**30.58**	**Andrastin B**				

Compounds detected by negative electrospray ionization (ESI-) are in **bold**. **^a^** R_T_ = retention time, dpi = days post inoculation.

**Table 3:** Secondary metabolites detected in synnemata that pierced the epicarp of apples infected with the Pe∆*brlA* strain (30 dpi).

Molecular Formula	^12^C *m*/*z* (Da)	^a^ R_T_ (min)	Proposed Identification	Molecular Formula	^12^C *m*/*z* (Da)	^a^ R_T_ (min)	Proposed Identification
C_15_H_20_O_4_	265.14412	15.91	Expansolide C/D	**C_28_H_38_O_8_**	**501.24786**	**29.57**	
C_15_H_20_O_4_	265.14415	18.49	Expansolide C/D	**C_28_H_40_O_6_**	**471.27434**	**39.48**	**Andrastin C**
C_16_H_18_N_2_O_2_	271.14496	7.62	Clavicipitic acid	**C_28_H_40_O_7_**	**487.26898**	**30.58**	**Andrastin B**
C_16_H_26_N_2_O_4_S_2_	375.14202	22.02		C_29_H_33_N_5_O_6_	548.25180	14.66	
C_17_H_22_O_5_	307.15471	27.39	Expansolide A/B	C_32_H_34_N_4_O_3_	523.27152	31.84	Communesin D
C_17_H_22_O_5_	307.15504	30.19	Expansolide A/B	C_32_H_36_N_2_O_4_	513.27635	39.10	Chaetoglobosin J or Prochaetoglobosin III
C_18_H_16_N_2_O_2_	293.12915	17.74		C_32_H_36_N_2_O_5_	529.27029	26.79	Chaetoglobosin B/G
C_18_H_18_N_2_O_2_	295.14479	14.61		C_32_H_36_N_2_O_5_	529.26757	29.56	Chaetoglobosin B/G
**C_19_H_38_O_6_**	**361.25819**	**38.19**		C_32_H_36_N_2_O_5_	529.27077	30.51	Chaetoglobosin B/G
**C_22_H_20_N_3_O**	**341.15380**	**38.56**		C_32_H_36_N_2_O_5_	529.27067	33.36	Chaetoglobosin A
**C_22_H_20_N_3_O**	**341.15368**	**39.29**		C_32_H_36_N_2_O_5_	529.26769	35.49	Chaetoglobosin B/G
C_22_H_23_N_5_O_2_	390.19390	15.09	Roquefortine C	C_32_H_36_N_2_O_5_	529.27063	36.74	Chaetoglobosin C
C_22_H_25_N_5_O_2_	392.20913	9.99	Roquefortine D	C_32_H_36_N_2_O_5_	529.27056	37.41	Chaetoglobosin B/G
C_23_H_24_N_2_O_6_	425.17179	26.92		C_32_H_36_N_2_O_6_	545.26332	27.60	Putative cytochalasan
**C_24_H_26_N_2_O_6_**	**437.17091**	**33.79**		C_32_H_36_N_2_O_6_	545.26241	29.23	Putative cytochalasan
C_26_H_30_N_4_	399.25568	19.94	Communesin K	C_32_H_36_N_2_O_6_	545.26288	30.57	Putative cytochalasan
C_26_H_30_N_4_O	415.25034	14.62	Communesin I	C_32_H_36_N_2_O_6_	545.26294	32.48	Putative cytochalasan
C_26_H_30_N_4_O	415.25033	18.43	Communesin I	C_32_H_36_N_4_O_2_	509.29257	34.95	Communesin B
C_26_H_40_O_6_	449.28939	29.93		C_32_H_38_N_2_O_4_	515.28895	35.59	Putative cytochalasan
C_27_H_30_N_4_O_2_	443.24564	15.65	Communesin E	C_32_H_38_N_2_O_4_	515.28890	37.93	Putative cytochalasan
C_28_H_30_N_4_O_3_	471.23997	19.56	Com470	C_32_H_38_N_2_O_4_	515.28909	38.15	Putative cytochalasan
C_28_H_32_N_4_O	441.26589	17.14	Communesin F	C_32_H_38_N_2_O_5_	531.28990	22.76	Putative cytochalasan
C_28_H_32_N_4_O_2_	457.26116	23.65	Communesin A	C_32_H_38_N_2_O_5_	531.28431	23.91	Putative cytochalasan
**C_28_H_38_O_7_**	**485.25405**	**35.32**		C_32_H_38_N_2_O_5_	531.28354	28.02	Chaetoglobosin E
C_28_H_38_O_7_	487.27063	36.21	Andrastin A	C_32_H_38_N_2_O_5_	531.28338	31.56	Penochalasin
**C_28_H_38_O_8_**	**501.24792**	**27.70**		C_32_H_38_N_2_O_6_	547.27864	23.19	Putative cytochalasan
**C_28_H_38_O_8_**	**501.24792**	**28.44**		C_32_H_38_N_2_O_6_	547.27841	25.47	Putative cytochalasan

Compounds detected by negative electrospray ionization (ESI-) are in **bold**. **^a^** R_T_ = Retention time.

**Table 4:** Differential expressed genes (DEG) involved in fungal development.

	*Penicillium expansum* d1 Strain Gene ID	Protein Name	Log_2_ Fold Change PeΔ*brlA* vs. WT	Adjusted *p*-Value	Putative Role
**Regulation of Development**	PEXP_029020	AbaA	−3.62	4.07 × 10^−11^	Transcription factor
	PEXP_077410	WetA	−3.82	1.02 × 10^−10^	DNA-binding transcription factor
	PEXP_085800	Axl2	−2.51	5.36 × 10^−11^	Phialide morphogenesis regulatory protein
	PEXP_040110	PhiA	1.83	1.73 × 10^−5^	Phialide development protein
	PEXP_003940	VadA	−2.83	1.37 × 10^−9^	Spore-specific regulator
	PEXP_102520	DnjA	1.33	2.75 × 10^−7^	DnaJ familly chaperone
	PEXP_064110	MedA	1.13	3.51 × 10^−5^	Temporal modifier of developmental
	PEXP_050580	PpoC	1.29	6.05 × 10^−6^	*psi*-Producing oxygenase
**Hydrophobins**	PEXP_062290	RodA	−13.00	1.07 × 10^−18^	Rodlet A, Hydrophobic protein
	PEXP_020490	RodB/DewB	−11.4	1.43 × 10^−16^	Rodlet B, Hydrophobic protein
	PEXP_071760	DewC	−0.546	2.14 × 10^−1^	
	PEXP_043320	DewD	−5.62	5.94 × 10^−13^	
	PEXP_098360	DewE	−0.906	2.41 × 10^−3^	
**Pigmentation** **DHN-Melanin Like Pigment**	PEXP_096630	Alb1	−12.2	6.41 × 10^−17^	Putative polyketide synthase
	PEXP_097170	Arp1	−8.64	1.31 × 10^−15^	Putative protein-Conidial pigmentation
	PEXP_097180	Arp2	−8.34	1.13 × 10^−13^	HN reductase
	PEXP_097190	Ayg1	−6.57	9.55 × 10^−13^	
	PEXP_097110	Abr1	−6.57	1.58 × 10^−12^	Multicopper oxidase
**Trehalose Biosynthesis**	PEXP_050560	Ccg-9	−5.04	2.47 × 10^−6^	Clock-controlled gene 9
**Kinase**	PEXP_066390	Gin4	−5.00	2.02 × 10^−12^	Localization and function of septins
**Velvet Protein Family**	PEXP_092360	VeA	0.89	1.05 × 10^−5^	Global transcription factor
	PEXP_065290	VelB	−0.53	1.01 × 10^−3^	Velvet-like protein B
	PEXP_009420	VelC	0.43	3.66 × 10^−4^	Regulator of sexual development
	PEXP_042660	LaeA	−0.44	5.39 × 10^−3^	Putative methyltransferase
	PEXP_076870	VosA	−0.98	2.35 × 10^−4^	Multifunctional regulator of development

**Table 5:** Differentially expressed genes in PeΔ*brlA* strain coding for backbone enzymes involved in secondary metabolite biosynthesis.

	*Penicillium expansum* Strain d1 Gene ID	Biosynthetic Gene Cluster	Log_2_ Fold Change PeΔ*brlA* vs. WT	Adjusted *p*-Value
**DMATS (Dimethylallyl tryptophane synthase)**	PEXP_030140	Roquefortine C	1.68	1.40 × 10^−5^
	PEXP_030510	Communesins	−4.64	3.08 × 10^−10^
	PEXP_058590	-	−1.31	2.72 × 10^−8^
**PKS** **(Polyketide synthase)**	PEXP_006700	-	−4.51	5.61 × 10^−15^
	PEXP_028920	-	4.51	1.77 × 10^−4^
	PEXP_030540	Communesins	−1.76	5.50 × 10^−7^
	PEXP_037250	-	−2.14	4.85 × 10^−8^
	PEXP_063170	-	2.73	1.29 × 10^−2^
	PEXP_076200	-	−1.44	4.74 × 10^−5^
	PEXP_094460	Patulin	−1.03	1.21 × 10^−5^
	PEXP_094770	-	1.12	3.62 × 10^−3^
	PEXP_095510	-	−2.89	7.63 × 10^−8^
	PEXP_096630	Pigment	−12.2	4.72 × 10^−20^
	PEXP_097790	-	1.52	4.63 × 10^−3^
	PEXP_099180	-	−1.81	4.23 × 10^−10^
	PEXP_102410	-	−1.92	3.65 × 10^−10^
**NRPS** **(Non ribosomal peptide synthetase)**	PEXP_012360	-	−1.35	7.72 × 10^−8^
	PEXP_015170	Fungisporins	1.71	1.34 × 10^−7^
	PEXP_018960	-	−8.55	2.90 × 10^−11^
	PEXP_029660	-	−2.97	7.36 × 10^−10^
	PEXP_030090	Roquefortine C	0.99	2.57 × 10^−3^
	PEXP_055140	-	1.55	1.17 × 10^−8^
	PEXP_095540	-	−3.46	7.65 × 10^−9^
	PEXP_096300	-	2.95	1.96 × 10^−11^
	PEXP_104890	-	−1.1	2.04 × 10^−4^
**Hybrid PKS/NRPS**	PEXP_008740	-	1.02	1.41 × 10^−5^
	PEXP_074060	Chaetoglobosins	2.17	5.15 × 10^−10^
**NRPS-like**	PEXP_045260	-	3.89	8.31 × 10^−7^
	PEXP_050450	-	1.03	2.53 × 10^−7^
	PEXP_060620	-	2.11	5.21 × 10^−11^
	PEXP_072870	-	−2.05	6.25 × 10^−8^
	PEXP_080590	-	−1.4	1.68 × 10^−8^
	PEXP_082750	-	1.18	7.81 × 10^−9^
	PEXP_095480	-	1.43	2.51 × 10^−10^
Terpene cyclase	PEXP_043150	-	−2.68	1.19 × 10^−4^

**Table 6:** Putative chaetoglobosin gene cluster.

*Penicillium expansum* Strain d1	*Chaetomium globosum* Strain CBS 148.51	% Identity/Similarity	Log_2_ Fold Change PeΔ*brlA* vs. WT	Putative Function
PEXP_073960	CHGG_01242.1/**CHGG_05285**	48/64; **53/70**	1.84	CYP450
PEXP_073970	CHGG_01240/**CHGG_05283**	41/63; **64/79**	2.07	Enoyl reductase
PEXP_073980	CHGG_01241/**CHGG_05282**	48/64; **65/80**	1.68	Hypothetical protein
PEXP_073990	CHGG_01240/**CHGG_05283**	47/65; **39/54**	1.89	Enoyl reductase
PEXP_074000	CHGG_01244	41/55;	2.08	Hypothetical protein
PEXP_074010	CHGG_01243/**CHGG_05281**	47/66; **53/71**	1.82	CYP P450
PEXP_074020	CHGG_05287	31/49;	1.82	Transcription factor *
PEXP_074030	CHGG_01245/**CHGG_05284**	47/62; **43/59**	1.95	Short-chain dehydrogenase
PEXP_074040	CHGG_01242.2/**CHGG_05280**	38/53; **48/65**	1.84	FAD-dependent oxidoreductase
PEXP_074050	CHGG_01246/**CHGG_05287**	54/70; **54/72**	1.97	Alpha/beta hydrolase
PEXP_074060	CHGG_01239/**CHGG_05286**	43/61; **52/68**	2.17	PKS-NRPS

* Homologous with cytochalasin pathway-specific TF CcsR (ACLA_078640) in *Aspergillus clavatus* [67]. In **bold** a second chaetoglobosin gene cluster is present in *Chaetomium globosum* genome.

## Supplementary Materials

The following are available online at https://www.mdpi.com/1422-0067/21/18/6660/s1, Figure S1: Morphological aspect of *Penicillium expansum* wild type NRRL 35695 and the null mutant Pe∆*brlA* strains, Figure S2: Rot growth rates obtained from Golden Delicious apples infected with *Penicillium expansum* wild type NRRL 35695 and the null mutant Pe∆*brlA* strains, Figure S3: Morphological aspect of (A) Null mutant PeΔ*brlA* strain and (B) *Penicillium expansum* wild type NRRL 35695 strain grown in a minimal media supplemented with galactose, Figure S4: Relative gene expression of the putative chaetoglobosin biosynthetic gene cluster (PEXP_073960-PEXP_074060) in *Penicillium expansum* wild type NRRL 35695 and the null mutant Pe∆*brlA* strains, Figure S5: Double-joint PCR reaction, Figure S6: PCR amplification of *Penicillium expansum* wild type NRRL 35695 (WT) and null mutant Pe∆*brlA* strains, Figure S7: Genome walking (GW) analyses of genomic DNA of Penicillium expansum wild type NRRL 35695 and null mutant PeΔbrlA strains, Figure S8: Validation by quantitative real-time PCR analysis, Table S1: MS/MS spectra of secondary metabolites detected from *Penicillium expansum* wild type NRRL 35695 after culture on labeled wheat grains, Table S2: MS/MS spectra of the specific secondary metabolites only detected in the null mutant Pe∆*brlA* strain after culture on labeled wheat grains, Table S3: Primers used in qPCR for analysis of putative chaetoglobosin gene clusters, Table S4: *Penicillium expansum* genes orthologous to genes significantly down-regulated (Log2 fold change < −3) in *Penicillium digitatum* Pd∆*brlA* strain [38], Table S5: *Penicillium expansum* genes orthologous to genes significantly up-regulated (Log2 fold change > 2) in *Penicillium digitatum* Pd∆*brlA* strain [38], Table S6: Eighteen orthologous genes similarly regulated to *wetA* in *Penicillium rubens* Δ*brlA* [49] and *Penicillium expansum* Δ*brlA*, Table S7: Fifty-nine orthologous genes similarly down-regulated to *abaA* in *Penicillium rubens*, Table S8: Primers used in the construction and the validation of the null mutant Pe∆*brlA* strain. Table S9: List of differentially expressed genes.

## Abbreviations

APAM	Apple Puree Agar Medium
bp	base pair
CRP	Central Regulatory Pathway
Com	Communesin
CYA	Czapek Yeast extract Agar
DEG	Differential Expressed Genes
DHN	1,8-Dihydroxynaphthalene
dpi	day post inoculation
GEO	Gene Expression Omnibus
HPLC	High Performance Liquid Chromatography
LBG	Locus Bean Gum
LC-HRMS	Liquid Chromatography-High Resolution Mass Spectrometry
L_hgu_	hyphal growth unit Length
MEA	Malt Extract agar
PDA	Potato Dextrose Agar
Rt	Retention time, min
SM	Secondary Metabolite
TF	Transcription Factor
WT	Wild Type
