# Supplementary material for: The brlA Gene Deletion Reveals That Patulin Biosynthesis Is Not Related to Conidiation in Penicillium expansum
Source: Int J Mol Sci. 2020 Sep 11;21(18):6660. doi: 10.3390/ijms21186660 (PMC7555563; doi:10.3390/ijms21186660)
Supplement: Supplementary file 1 [file ijms-21-06660-s001.zip › Supplementary Materials/ijms-909423 Supp. Materials.pdf]

IJMS

Supplementary Materials

The *brlA* gene deletion reveals that patulin biosynthesis is not related to conidiation in *Penicillium expansum*

Chrystian Zetina-Serrano [1], Ophélie Rocher [1], Claire Naylies [1], Yannick Lippi [1], Isabelle P. Oswald [1], Sophie Lorber [1] and Olivier Puel [1,*]

[1] Toxalim (Research Centre in Food Toxicology), Université de Toulouse, INRAE, ENVT, INP-Purpan, UPS, Toulouse, France; Chrystian-Del-Carmen.Zetina-Serrano@inrae.fr (C.Z.); ophelie.rocher@inrae.fr (O.R.); claire.naylies@inrae.fr (C.N.); yannick.lippi@inrae.fr (Y.L.); isabelle.oswald@inrae.fr (I.O.); sophie.lorber@inrae.fr (S.L.); olivier.puel@inrae.fr (O.P.)

* Correspondence: olivier.puel@inrae.fr; Tel.: +33 582 066 336

Table of contents

Supplementary Figure 1. Morphological aspect of *Penicillium expansum* wild type NRRL 35695 and the null mutant PeΔ*brlA* strains. 33

Supplementary Figure 2. Rot growth rates obtained from Golden Delicious apples infected with *Penicillium expansum* wild type NRRL 35695 and the null mutant PeΔ*brlA* strains and incubated at 25 °C for 14 days in the dark. 44

Supplementary Figure 3. Morphological aspect of A) Null mutant PeΔ*brlA* strain and B) *Penicillium expansum* wild type NRRL 35695 strain grown in a minimal media supplemented with galactose 55

Supplementary Table 1. MS/MS spectra of secondary metabolites detected from *Penicillium expansum* wild type NRRL 35695 after culture on labeled wheat grains. 66

Supplementary Table 2. MS/MS spectra of the specific secondary metabolites only detected in the null mutant PeΔ*brlA* strain after culture on labeled wheat grains. 1010

Supplementary Table 3. Primers used in qPCR for analysis of putative chaetoglobosin gene clusters. 1212

Supplementary Figure 4. Relative gene expression of the putative chaetoglobosin biosynthetic gene cluster (PEXP_073960-PEXP_074060) in *Penicillium expansum* wild type NRRL 35695 and the null mutant PeΔ*brlA* strains 1313

Supplementary Table 4. *Penicillium expansum* genes orthologous to genes significantly down-regulated (Log_2 fold change < -3) in *Penicillium digitatum* PdΔ*brlA* strain [38]. 1414

Supplementary Table 5. *Penicillium expansum* genes orthologous to genes significantly up-regulated (Log_2 fold change > 2) in *Penicillium digitatum* PdΔ*brlA* strain [38]. 1818

Supplementary Table 6. Eighteen orthologous genes similarly regulated to *wetA* in *Penicillium rubens* Δ*brlA* [49] and *Penicillium expansum* Δ*brlA* 2020

Supplementary Table 7. Fifty-nine orthologous genes similarly down-regulated to *abaA* in *Penicillium rubens* 2121

Construction and Characterization of the null mutant PeΔ*brlA* strain 2323

Creation of a Linear Transformation Cassette using Double-Joint PCR 2323

Supplementary Table 8. Primers used in the construction and the validation of the null mutant PeΔ*brlA* strain. 2424

Supplementary Figure 5. Double-joint PCR reaction 2525

Validation of the Null Mutant PeΔbrlA strain 2626

Polymerase Chain Reaction (PCR) and Genome Walking 2626

Supplementary Figure 6. PCR amplification of *Penicillium expansum* wild type NRRL 35695 (WT) and null mutant PeΔ*brlA* strains. 2727

Supplementary Figure 7. Genome walking (GW) analyses of genomic DNA of *Penicillium expansum* wild type NRRL 35695 and null mutant PeΔ*brlA* strains. .... 27~~27~~

Quantitative real-time PCR Validation .... 28~~28~~

Supplementary Figure 8. Validation by quantitative real-time PCR analysis. The expression of the *brlA* gene was evaluated in *Penicillium expansum* wild type NRRL 35695 (WT) and in the null mutant PeΔ*brlA* strains. .... 29~~29~~

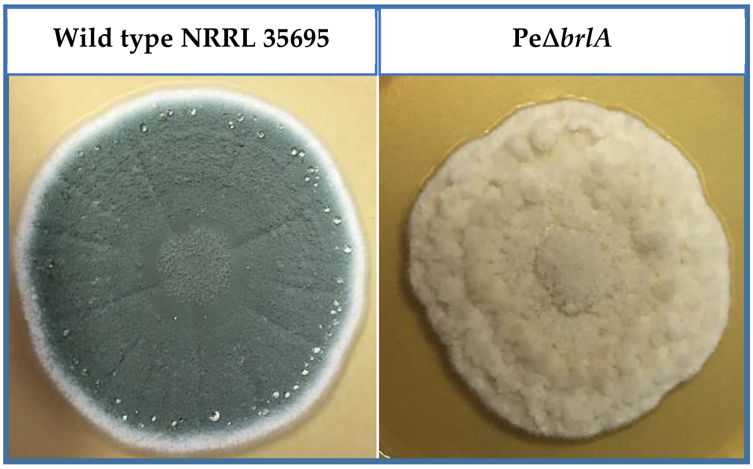

Supplementary Figure 1. Morphological aspect of *Penicillium expansum* wild type NRRL 35695 and the null mutant PeΔ*brlA* strains. The strains were grown on MEA at 25 °C in the dark for seven days.

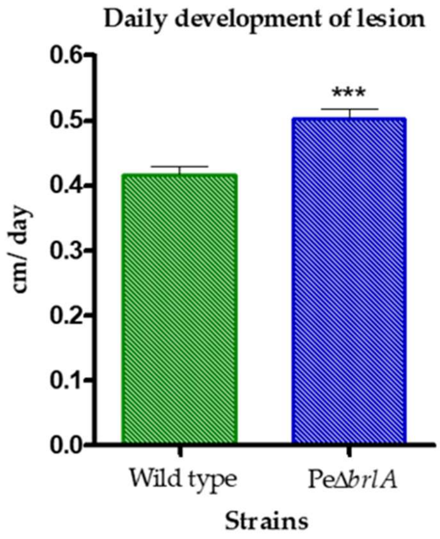

Supplementary Figure 2. Rot growth rates obtained from Golden Delicious apples infected with *Penicillium expansum* wild type NRRL 35695 and the null mutant PeΔ*brlA* strains and incubated at 25 °C for 14 days in the dark. The graph shows the mean ± standard error of the mean (SEM) from nine biological replicates. *** p-value < 0.001.

A) B)

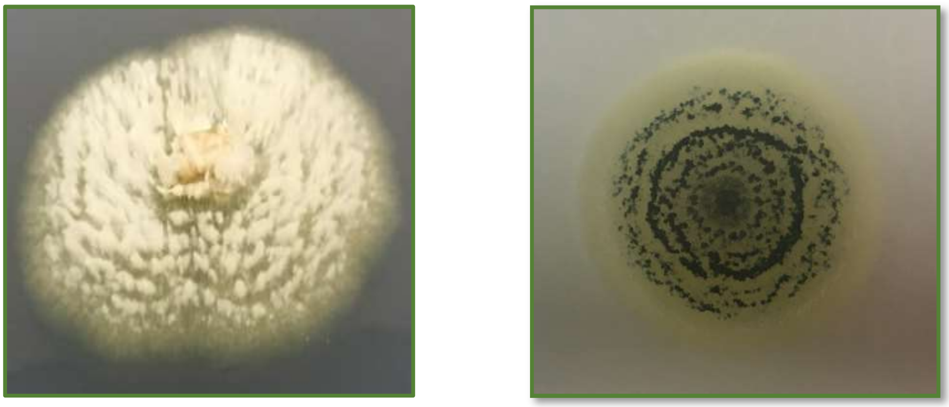

Supplementary Figure 3. Morphological aspect of A) Null mutant PeΔ*brlA* strain and B) *Penicillium expansum* wild type NRRL 35695 strain grown in a minimal media supplemented with galactose and incubated for seven days at 25 °C in the dark. This medium promoted the development of coremia or synnemata.

Supplementary Table 1. MS/MS spectra of secondary metabolites detected from *Penicillium expansum* wild type NRRL 35695 after culture on labeled wheat grains.

Molecular formula	Identifier [a]	^{12}C *m/z* (Da)	^{13}C *m/z* (Da)	^{13}C/^{15}N *m/z* (Da)	*m/z* of major fragment ions
**$C_7H_6O_4$**	**Pexp_153.019_3.59**	**153.01919**	**160.04256**	**156.02997**	**153→ 125, 109(100), 97**
C_7H_8O	Pexp_109.065_7.11	109.06509	116.08844	112.07483	-
$C_7H_8O_2$	Pexp_125.060_6.79	125.05998	132.08346	129.07332	125→ 106(100), 97, 95, 83, 81, 79, 69
$C_7H_8O_3$	Pexp_141.054_3.80	141.05493	148.07869	144.06492	141→ 123, 113(100), 110, 95, 79, 69, 55
$C_7H_8O_4$	Pexp_157.049_2.67	157.04990	164.07348	161.06366	157→ 139(100), 111, 97, 83
$C_7H_{10}O_3$	Pexp_143.070_4.52	143.07061	150.09415	147.08401	143→ 125, 113(100), 107, 97, 79
$C_{10}H_{17}NO_5$	Pexp_232.118_8.83	232.11872	242.15222	238.13252	232→ 214, 185, 157, 119 (100), 112, 101, 86
$C_{12}H_{14}O_3$	Pexp_207.101_9.65	207.10110	219.14115	213.12114	207→ 189(100), 179, 174, 171, 161, 142, 137
$C_{13}H_{14}O_5$	Pexp_251.091_21.70	251.09108	264.13455	258.11474	251→ 233(100), 215, 198, 167, 143
$C_{15}H_{18}N_2$	Pexp_227.155_6.44	227.15514	242.20521	237.17554	227→ 210(100), 198, 171, 154, 130
$C_{15}H_{19}NO_6$	Pexp_310.129_12.78	310.12939	325.17984	319.15312	310→ 292 (100), 264, 240, 186, 158, 140, 107
$C_{15}H_{19}NO_6$	Pexp_310.129_14.80	310.12964	325.17835	319.14477	310→ 292 (100), 264, 240, 185, 158, 140, 107
$C_{15}H_{20}O_4$	Pexp_265.144_15.91	265.14412	280.19431	273.17072	265→ 247, 229, 219(100), 205, 201, 191, 151, 135, 107
$C_{15}H_{20}O_4$	Pexp_265.144_16.91	265.14410	280.19364	273.17064	265→ 247, 229, 219(100), 205, 201, 191 151, 135, 107
$C_{15}H_{20}O_4$	Pexp_265.144_18.49	265.14415	280.19430	273.17081	265→ 247, 229, 219(100), 205, 201, 191 151, 135, 107
$C_{15}H_{20}O_4$	Pexp_265.144_19.35	265.14414	280.19429	273.17082	265→ 247, 229, 219(100), 205, 201, 191 151, 135, 107
$C_{16}H_{18}N_2O_2$	Pexp_271.144_7.62	271.14496	287.19861	281.16599	271→ 254(100), 225, 215, 168
$C_{16}H_{18}N_2O_2$	Pexp_271.144_8.45	271.14467	287.19873	281.16576	271→ 254(100), 225, 215, 168
$C_{16}H_{26}N_2O_4S_2$	Pexp_375.142_22.02	375.14202	391.19584	385.16297	375→ 357, 347, 222, 178, 154(100),
$C_{17}H_{22}O_5$	Pexp_307.154_27.39	307.15471	324.21155	316.18482	307→ 265(100), 247, 229, 219, 211, 201, 135
$C_{17}H_{22}O_5$	Pexp_307.155_30.19	307.15504	324.21163	316.18515	307→ 265(100), 247, 229, 219, 211, 201, 135
$C_{18}H_{16}N_2O_2$	Pexp_293.129_13.58	293.12911	311.18978	305.15587	293→ 265(100), 248, 237, 222, 120
$C_{18}H_{16}N_2O_2$	Pexp_293.129_17.74	293.12915	311.18970	305.15668	293→ 265(100), 248, 237, 222, 120
$C_{18}H_{16}N_2O_3$	Pexp_309.124_14.76	309.12421	327.18455	320.14798	309→ 292 (100), 267, 249, 231

$C_{18}H_{18}N_2O_2$	Pexp_295.144_14.61	295.14479	313.20540	307.17251	295→ 267(100), 250, 239, 222, 120
$C_{18}H_{31}NO_7$	Pexp_374.216_23.72	374.21663	392.27652	385.24644	374→ 356(100), 204, 186, 158
$C_{19}H_{16}N_2O_2$	Pexp_305.129_32.50	305.12911	324.19310	316.15289	305→ 277(100), 264, 262, 205, 187
$C_{19}H_{16}N_2O_2$	Pexp_305.129_33.17	305.12933	324.19301	317.15701	305→ 277(100), 264, 262, 205, 187
$C_{19}H_{16}N_2O_4$	Pexp_337.119_13.73	337.11909	356.18283	349.14659	337→ 319, 309(100), 296, 294, 280, 252, 237, 203, 121
$C_{19}H_{16}N_2O_4$	Pexp_337.119_15.20	337.11916	356.18294	349.14676	337→ 319, 309(100), 296, 280, 237, 203, 121
$C_{19}H_{21}NO_7$	Pexp_376.139_17.45	376.13901	395.20280	387.16962	376→ 233, 207(100), 189
$C_{19}H_{21}NO_7$	Pexp_376.139_18.65	376.13904	395.20262	387.16966	376→ 233, 207(100), 189
**$C_{19}H_{38}O_6$**	**Pexp_361.258_37.23**	**361.25871**	**380.32251**	**371.29242**	**361→ 343, 325, 283, 273, 257(100), 157**
**$C_{19}H_{38}O_6$**	**Pexp_361.258_38.19**	**361.25819**	**380.32253**	**371.29252**	**361→ 343, 325, 299, 283, 257(100), 157**
$C_{20}H_{18}N_2O_2$	Pexp_319.145_35.48	319.14588	339.21182	332.17585	319→ 291(100), 278, 262, 250, 234, 205, 201, 188, 132
$C_{20}H_{18}N_2O_2$	Pexp_319.145_36.44	319.14502	339.21173	332.17596	319→ 291(100), 278, 260, 250, 234, 205, 201, 188, 160, 132
$C_{20}H_{21}NO_9$	Pexp_420.128_18.38	420.12857	440.19571	432.16272	420→ 251(100), 233
$C_{20}H_{21}NO_9$	Pexp_420.128_21.20	420.12858	440.19605	432.16318	420→ 251(100), 233
$C_{20}H_{26}O_8$	Pexp_395.171_13.61	395.17142	415.23827	406.20763	395→ 251(100), 233
$C_{22}H_{23}N_5O_2$	Pexp_390.193_15.09	390.19390	412.26737	406.21627	390→ 334, 322(100), 198, 193
$C_{22}H_{25}N_5O_2$	Pexp_392.209_9.99	392.20913	414.28326	408.22845	392→ 336, 324(100), 198, 195
$C_{23}H_{24}N_2O_6$	Pexp_425.171_26.92	425.17179	448.24874	439.20611	425→ 407, 383, 365, 288(100), 260
**$C_{24}H_{26}N_2O_6$**	**Pexp_437.170_33.79**	**437.17091**	**461.25116**	**451.20575**	**437→ 395, 377(100), 333, 286**
$C_{26}H_{30}N_4$	Pexp_399.255_19.94	399.25568	425.34268	417.29076	399→ 382, 365, 241(100), 224, 185, 159, 144, 130
$C_{26}H_{30}N_4O$	Pexp_415.250_14.62	415.25034	441.33736	433.28593	415→ 398, 357, 343, 257(100), 240, 199, 185, 159
$C_{26}H_{30}N_4O$	Pexp_415.250_18.43	415.25033	441.33739	433.28576	415→ 397, 257(100), 240, 228, 224, 201, 159, 130
$C_{26}H_{40}O_6$	Pexp_449.289_29.93	449.28939	475.37674	463.33658	449→ 431(100), 386, 384, 221, 211, 203, 179, 166
$C_{27}H_{30}N_4O_2$	Pexp_443.245_15.65	443.24564	470.33554	461.28105	443→ 385, 371(100), 201, 185, 171, 154
$C_{28}H_{30}N_4O_3$	Pexp_471.239_19.56	471.23997	499.33402	490.27887	471→ 399, 371, 201, 199(100), 171, 159, 154
$C_{28}H_{31}N_5O_5$	Pexp_518.241_16.36	518.24108	546.33497	538.27672	518→ 500, 462, 450(100), 321
$C_{28}H_{31}N_5O_5$	Pexp_518.240_17.21	518.24092	546.33488	538.27706	518→ 500, 462, 450(100), 402, 390, 326, 210
$C_{28}H_{32}N_4O$	Pexp_441.265_17.14	441.26589	469.35970	460.30445	441→ 424(100), 385, 365, 239, 224, 185, 183

$C_{28}H_{32}N_4O_2$	Pexp_457.260_14.27	457.26097	485.35452	476.29968	457→ 440(100), 385, 257,255, 240, 210, 201, 185
$C_{28}H_{32}N_4O_2$	Pexp_457.260_14.97	457.26095	485.35394	476.29996	457→ 439, 385(100), 239, 185
$C_{28}H_{32}N_4O_2$	Pexp_457.261_23.65	457.26116	485.35429	476.29971	457→ 399, 385(100), 201, 185, 159, 144, 130
$C_{28}H_{32}N_4O_3$	Pexp_473.255_15.50	473.25566	501.34996	492.29429	473→ 456(100), 401, 397, 273, 271, 253, 244, 201, 199, 171
$C_{28}H_{36}N_4O_4$	Pexp_493.279_31.47	493.27971	521.37363	511.31495	-
$C_{28}H_{38}N_4O_5$	Pexp_511.290_18.01	511.29099	539.38447	529.32631	511→ 493, 465, 394, 346, 301, 265(100), 247, 219, 166
$C_{28}H_{38}N_4O_6$	Pexp_527.287_12.46	527.28755	555.38184	545.32412	527→ 281(100), 509, 481, 346, 301, 265, 247, 219, 182
**$C_{28}H_{38}O_7$**	**Pexp_485.254_35.32**	**485.25405**	**513.34696**	**500.30389**	**485→ 470, 457, 453(100), 425, 411, 409, 393**
$C_{28}H_{38}O_7$	Pexp_487.270_36.21	487.27063	515.36381	502.32065	487→ 427(100), 409, 395, 377, 243
**$C_{28}H_{38}O_8$**	**Pexp_501.247_27.70**	**501.24792**	**529.34203**	**516.29846**	**501→ 457, 441(100), 425, 410, 397, 373, 365,**
**$C_{28}H_{38}O_8$**	**Pexp_501.247_28.44**	**501.24792**	**529.34203**	**516.29846**	**501→ 457, 441(100), 425, 410, 397, 373, 365,**
**$C_{28}H_{38}O_8$**	**Pexp_501.247_29.57**	**501.24786**	**529.34194**	**516.29974**	**501→ 486, 469, 457, 441(100), 425, 397, 373, 366**
**$C_{28}H_{40}O_6$**	**Pexp_471.274_39.48**	**471.27434**	**499.36727**	**486.32419**	**471→ 456, 439, 411(100)**
**$C_{28}H_{40}O_7$**	**Pexp_487.268_30.58**	**487.26898**	**515.36247**	**502.31903**	**487→ 455, 427(100), 411, 395, 351,336**
$C_{29}H_{33}N_5O_6$	Pexp_548.251_15.42	548.25180	577.34902	568.28775	548→ 530, 518, 492, 480(100), 462, 402, 390, 356
$C_{30}H_{36}N_4O_2$	Pexp_485.292_16.36	485.29245	515.39378	505.33425	485→ 468(100), 429, 365, 239, 224. 222, 183, 185, 159, 144
$C_{31}H_{36}N_4O_2$	Pexp_497.290_31.66	497.29061	528.39421	517.33225	497→ 479, 425(100), 439, 257, 241, 185, 168, 159
$C_{32}H_{34}N_4O_3$	Pexp_523.271_31.84	523.27152	555.37917	543.30477	523→ 451, 423(100), 253, 199, 171, 159, 154,
$C_{32}H_{36}N_2O_4$	Pexp_513.276_39.10	513.27635	545.38309	532.32773	513→ 495(100), 477, 455, 430, 412, 384, 366, 250
$C_{32}H_{36}N_2O_5$	Pexp_529.270_25.21	529.27058	561.37837	548.32196	529→ 511(100), 493, 475, 382, 380, 362, 307, 185
$C_{32}H_{36}N_2O_5$	Pexp_529.270_26.71	529.27029	561.37834	548.32343	529→ 511(100), 493, 475, 382, 380, 362, 185
$C_{32}H_{36}N_2O_5$	Pexp_529.270_30.51	529.27077	561.37872	548.32190	529→ 511(100), 493, 469, 475, 483, 457, 451, 359, 333
$C_{32}H_{36}N_2O_5$	Pexp_529.270_33.98	529.27067	561.37843	548.32208	529→ 511(100), 501, 493, 483, 475, 465, 457, 431, 417, 400, 382
$C_{32}H_{36}N_2O_5$	Pexp_529.270_35.67	529.27092	561.37735	548.31076	529→ 511 (100), 493, 469, 382, 359, 331
$C_{32}H_{36}N_2O_5$	Pexp_529.270_36.41	529.27063	561.37820	548.32186	529→ 511(100), 493, 487, 483, 475, 469, 457, 400, 398, 382, 380, 364, 328, 310, 185
$C_{32}H_{36}N_2O_5$	Pexp_529.270_37.41	529.27056	561.37807	548.32181	529→511 (100), 493, 453, 380, 327, 185
**$C_{32}H_{36}N_2O_6$**	**Pexp_543.248_34.05**	**543.24836**	**575.35597**	**562.29931**	543→ 499, 481, 471, 445(100), 427, 375, 352, 248

$C_{32}H_{36}N_4O_2$	Pexp_509.292_36.01	509.29257	541.39875	530.3376	509→ 437(100), 451, 185
$C_{32}H_{36}N_4O_3$	Pexp_525.287_13.45	525.28735	557.39329	546.33239	525→ 507, 481, 467, 453(100), 409, 343, 340, 257, 240,225, 199, 185, 159
$C_{32}H_{36}N_4O_3$	Pexp_525.287_27.91	525.28705	557.39461	546.33215	525→ 508(100), 271, 273, 199
$C_{32}H_{38}N_4O_2$	Pexp_511.306_36.13	511.30684	543.41590	532.35205	511→ 439(100), 453, 185, 144
$C_{32}H_{38}N_4O_3$	Pexp_527.303_21.41	527.30304	559.41082	548.34834	527→ 509, 469, 453, 437(100), 275, 215
$C_{32}H_{38}N_4O_4$	Pexp_543.297_19.36	543.29789	575.40330	564.34345	543→ 525, 485, 471(100), 185, 168
$C_{32}H_{40}N_4O_2$	Pexp_513.322_36.57	513.32227	545.43031	533.36211	513→ 495, 455, 441(100), 257, 185
$C_{33}H_{38}N_4O_5$	Pexp_571.293_18.22	571.29324	604.40405	592.33802	571→ 553, 513, 499(100), 455, 397, 299
$C_{37}H_{42}N_4O_5$	Pexp_623.325_29.52	623.32511	660.44909	646.37781	623→ 565, 551(100), 507, 449, 437, 371, 354, 299

In bold the compounds detected by negative electrospray ionization (ESI-). a Secondary metabolites identifier (Pexp_*m/z*_RT). *m/z* = *m/z* ratio and RT= retention time.

Supplementary Table 2. MS/MS spectra of the specific secondary metabolites only detected in the null mutant PeΔ*brlA* strain after culture on labeled wheat grains.

Molecular formula	Identifier [a]	^{12}C *m/z* (Da)	^{13}C *m/z* (Da)	^{13}C/^{15}N *m/z* (Da)	*m/z* of major fragment ions
$C_{17}H_{23}N_3O_3$	Pexp_318.179_03.12	318.17943	335.23746	330.20149	318→ 301(100), 272, 258, 205, 188, 178, 170, 159, 132
$C_{17}H_{23}N_3O_3$	Pexp_318.180_25.05	318.18043	335.23716	330.20173	318→ 300, 272, 205(100), 188, 159, 141, 132
$C_{18}H_{35}N_3O_4$	Pexp_358.269_0.354	358.26901	376.32935	370.29051	358→ 340, 227(100), 199
$C_{19}H_{20}O_5$	Pexp_329.137_33.29	329.13738	348.20079	339.17092	329→ 311(100), 293, 285, 269, 258, 241, 229, 205, 177, 161
$C_{26}H_{32}O_8$	Pexp_473.215_9.67	473.21511	499.30281	487.26381	-
$C_{26}H_{32}O_8$	Pexp_473.215_10.64	473.21552	499.30112	487.26454	-
$C_{26}H_{32}O_8$	Pexp_473.214_34.14	473.21489	499.29541	487.25368	-
$C_{27}H_{29}N_5O_4$	Pexp_488.227_10.8	488.22783	515.30922	507.26058	488→ 470(100), 458, 420, 402, 390, 322
$C_{27}H_{29}N_5O_4$	Pexp_488.227_11.3	488.22766	515.31807	507.26066	488→ 470(100), 458, 420, 402, 390, 375, 322
$C_{29}H_{27}N_5O_5$	Pexp_526.206_15.7	526.20693	555.30405	546.24399	526→ 508, 498, 458(100), 442, 430, 412, 390, 374, 334, 322, 216, 193
$C_{29}H_{27}N_5O_5$	Pexp_526.206_17.61	526.20689	555.29234	546.24884	526→ 508, 498, 480, 470, 458(100), 390, 334, 322, 204, 192
$C_{29}H_{31}N_5O_5$	Pexp_530.238_14.83	530.23837	559.33601	550.27623	530→ 512, 468, 462, 390(100), 338, 322, 276, 252, 208, 193
$C_{29}H_{31}N_5O_5$	Pexp_530.238_15.85	530.23812	559.33514	550.27487	530→ 512, 474, 468, 462, 390(100), 338, 322, 276, 193
$C_{29}H_{31}N_5O_5$	Pexp_530.238_16.68	530.23840	559.33720	550.27338	530→ 512, 474, 462(100), 402, 390, 338, 320, 270, 210, 193
$C_{29}H_{33}N_5O_6$	Pexp_548.248_11.36	548.24883	577.34601	568.28444	548→ 530, 504, 480, 474(100), 390, 356, 322, 263, 193
$C_{29}H_{33}N_5O_6$	Pexp_548.248_12.14	548.24860	577.34575	568.28418	548→ 530, 518, 480, 474(100), 468, 402, 390, 356, 322
$C_{32}H_{36}N_2O_5$	Pexp_529.268_23.19	529.26807	561.37549	548.32110	529→ 511(100), 493, 486, 465, 431, 417, 403, 380, 349, 331, 292, 185, 167, 159
$C_{32}H_{36}N_2O_5$	Pexp_529.268_32.43	529.26807	561.37583	548.3197	529→ 511(100), 501, 493, 483, 475, 429, 411, 398, 382, 362, 327, 185
$C_{32}H_{36}N_2O_5$	Pexp_529.268_35.49	529.26769	561.37490	548.31990	529→ 511(100), 493, 483, 475, 471, 465, 455, 417, 380, 362, 347, 327, 319, 185, 167
$C_{32}H_{36}N_2O_6$	Pexp_545.262_29.23	545.26241	577.37065	564.31731	-
$C_{32}H_{36}N_2O_6$	Pexp_545.262_30.57	545.26288	577.36827	564.31312	545→ 527(100), 509, 491, 481, 463, 449, 429, 411, 396, 360, 185
$C_{32}H_{36}N_2O_6$	Pexp_545.262_31.50	545.26281	577.36975	564.31459	-
$C_{32}H_{36}N_2O_6$	Pexp_545.262_32.48	545.26294	577.36998	564.31425	545→ 527(100), 509, 499, 491, 473, 445, 427, 365, 363, 345, 184
$C_{32}H_{38}N_2O_5$	Pexp_531.283_21.75	531.28384	563.39083	550.33694	531→ 513(100), 495, 477, 459, 429, 411, 397, 384, 313, 295, 267, 185
$C_{32}H_{38}N_2O_5$	Pexp_531.284_23.91	531.28431	563.39246	549.33473	531→ 513(100), 495, 477, 455, 397, 371, 331, 185, 165
$C_{32}H_{38}N_2O_5$	Pexp_531.283_28.02	531.28354	563.39229	550.33654	531→ 513(100), 495, 477, 485, 471, 467, 459, 445, 431, 399, 364, 331, 185
$C_{32}H_{38}N_2O_5$	Pexp_531.283_28.52	531.28340	563.39080	549.32886	531→ 513(100), 495, 477, 449, 413, 397, 371, 331, 309, 185, 165
$C_{32}H_{38}N_2O_5$	Pexp_531.283_31.56	531.28338	563.39064	550.33378	531→ 513(100) ,495, 485, 477, 467, 459, 455, 384, 349, 331, 185

$C_{32}H_{38}N_2O_6$	Pexp_547.278_23.19	547.27864	579.38570	566.32988	547→ 529(100), 511, 493, 483, 465, 349, 185
$C_{32}H_{38}N_2O_6$	Pexp_547.278_25.47	547.27841	579.38548	566.32988	547→ 529(100), 511, 493, 483, 465, 417, 327, 185
$C_{32}H_{38}N_2O_6$	Pexp_547.278_27.09	547.27842	579.38554	566.32965	547→ 529(100), 511, 493, 465, 483, 429, 475, 380, 185

[a] Secondary metabolite identifier (Pexp_*m/z*_R_T). *m/z* = *m/z* ratio and R_T= retention time.

Supplementary Table 3. Primers used in qPCR for analysis of putative chaetoglobosin gene clusters.

Primer name	Sequence	Ratio **(F/R)**
cheC_F	GACCAACCTTGGCATATA	300/300
cheC_R	TCGTCGATCTGTATGTAGAC	
cheB_F1	TCAAATCCTCACGCTATCT	300/900
cheB_R1	GCATGTGGGAGACTGATA	
cheA_F2	GAAGTGGGTTGTGGTAATG	900/300
cheA_R2	CAGTAGATCCCTCCAATATGTA	
cheD_F1	GAGGAACGCCTTCTGTAA	300/900
cheD_R1	GCGAGATGTTTGCACTTT	
cheF_F2	ACACTGCTGAGAAGTTCA	900/900 and 900/300
cheF_R2	GTTGCGTATCTCATTCACC	
cheG_F2	CTCCAGCGATGGTTCCT	300/900
cheG_R2	GCCTTGGCTGGATGATTG	
PEXP_073960_F2	TCCATTCTCTTAGTCTAGTGAC	900/900
PEXP_073960_R2	TTCAGATGCTGGTTAGGG	
PEXP_073970_F1	GGTGATTGTGCTGGTATTG	900/300
PEXP_073970_R1	GAGGTAGAAGTGGGTTCAT	
PEXP_073980_F1	GAACAACCAGCCATTGAG	900/300
PEXP_073980_R1	GTTAGGATCGCGGTAGAA	
PEXP_073990_F	GAGACGGTTCTCGTCTTTGG	300/300
PEXP_073990_R	ACACGATGGCCGAGAAG	
PEXP_074000_F	CCAACGTTCGCTATCCA	300/300
PEXP_074000_R	TGCGGACCTTGGTAATG	
PEXP_074010_F2	GCCACAAACGACATTGA	300/900
PEXP_074010_R2	CGGAACAACACCAAGAG	
PEXP_074020_F1	GGAAGCCGATCAGGATAA	300/300
PEXP_074020_R1	GCTGGTCAGGACTAGTTG	
PEXP_074030_F	TGAAGTCCTCGAACCAA	300/900
PEXP_074030_R	TTTGCTTGTTCAGGATGG	
PEXP_074040_F1	TCCTGTCGCCTCATTAT	900/300
PEXP_074040_R1	CACCGCTGTTCTTGATAG	
PEXP_074050_F1	CACTGACGAACCAGAAAG	300/300
PEXP_074050_R1	GTTGCACAGAGCGATAG	
PEXP_074060_F2	TAGAGTTGGGTCGTGGT	300/900
PEXP_074060_R2	CTCTCTCACTGTACATCTTGG	

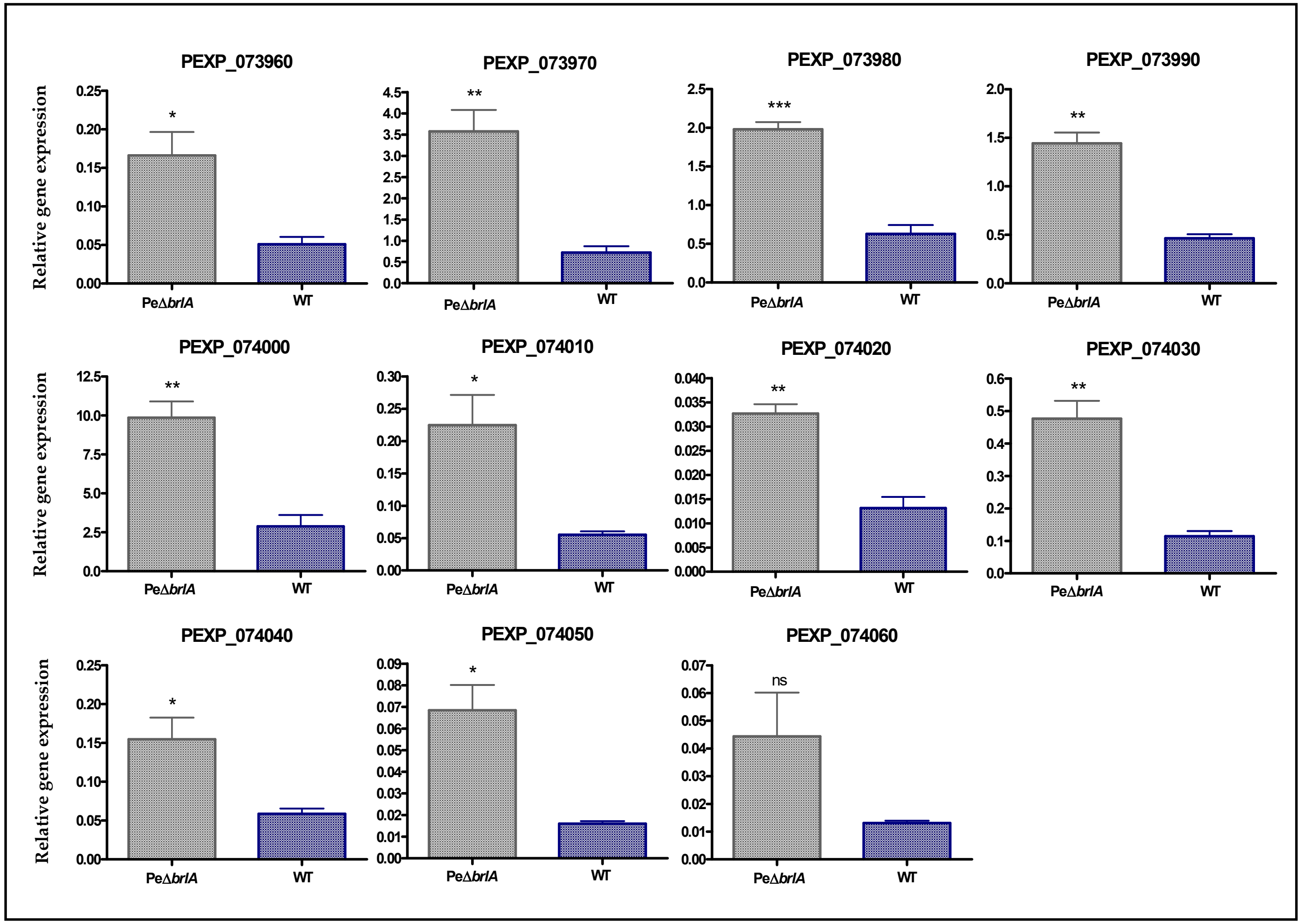

Supplementary Figure 4. Relative gene expression of the putative chaetoglobosin biosynthetic gene cluster (PEXP_073960-PEXP_074060) in *Penicillium expansum* wild type NRRL 35695 and the null mutant Pe*ΔbrlA* strains. The graphs show the mean ± standard error of the mean (SEM) from three biological replicates and the significant differences between the wild type and the null mutant Pe*ΔbrlA* strains. *p*-value *< 0.05; **< 0.01; ***< 0.001. ns = no significant.

Supplementary Table 4. *Penicillium expansum* genes orthologous to genes significantly down-regulated (Log2 fold change < -3) in *Penicillium digitatum* PdΔ*brlA* strain [38].

Penicillium digitatum PHI26 strain Gene ID	*Penicillium digitatum* GenBank Accession number	Log2 Fold Change PdΔ*brlA* Vs WT[1*]	% Positives	*Penicillium expansum* strain d1 Gene ID	Log2 fold change PeΔ*brlA* Vs WT[2]	Adjusted *p*-value
GI:425772521	EKV10922.1	-8.5	98.07	PEXP_097180	-8.34	1.13E-13
GI:425776459	EKV14676.1	-8.4	92.95	PEXP_020490	-11.4	1.43E-16
GI:425767953	EKV06503.1	-8.3	81.25	PEX2_067760	ns	-
GI:425772520	EKV10921.1	-8.1	95.19	PEXP_097170	-8.64	1.15E-15
GI:425779884	EKV17912.1	-7.9	88.53	PEXP_044610	-6.44	1.95E-03
GI:425765865	EKV04510.1	-7.6	74.22	PEXP_098690	-10.6	2.53E-18
GI:425772463	EKV10864.1	-7.2	87.2	PEXP_008790	-6.68	2.81E-14
GI:425767228	EKV05802.1	-7.0	ns	-	-	-
GI:425769821	EKV08303.1	-6.9	93.79	PEXP_020490	-11.4	1.43E-16
GI:425781523	EKV19483.1	-6.9	80.16	PEXP_033790	-8.30	8.53E-16
GI:425775975	EKV14214.1	-6.8	86.77	PEXP_062310	-10.9	7.29E-16
GI:425773118	EKV11490.1	-6.8	92.02	PEXP_001060	-9.51	1.43E-16
GI:425776321	EKV14543.1	-6.7	84.95	PEXP_075490	-2	6.55E-08
GI:425770425	EKV08898.1	-6.7	93.33	PEXP_052120	-10.1	8.09E-17
GI:425775531	EKV13796.1	-6.7	98.43	PEXP_049260	-6.21	1.07E-14
GI:425770751	EKV09215.1	-6.6	91.34	PEXP_004280	ns	-
GI:425781932	EKV19866.1	-6.6	95.51	PEXP_018490	-10.7	2.61E-16
GI:425775976	EKV14215.1	-6.5	96.95	PEXP_062290	-13.0	1.07E-18
GI:425774025	EKV12348.1	-6.5	78.85	PEXP_011470	-7.65	1.10E-14
GI:425765801	EKV04449.1	-6.5	87.60	PEXP_074900	-7.92	1.14E-14
GI:425779486	EKV17537.1	-6.4	97.28	PEXP_000200	-5.57	3.94E-13
GI:425774428	EKV12735.1	-6.4	66.67	PEXP_014810	-6.15	8.11E-15
GI:425773468	EKV11821.1	-6.3	62.31	PEXP_108060	-8.35	1.14E-14
GI:425769509	EKV08001.1	-6.3	97.23	PEXP_027310	ns	-

GI:425775526	EKV13791.1	-6.2	96.63	PEXP_049320	-1.18	3.91E-07
GI:425770516	EKV08985.1	-6.2	96.40	PEXP_092210	-4.28	1.47E-13
GI:425781930	EKV19864.1	-6.1	95.29	PEXP_018530	-7.61	1.31E-15
GI:425780307	EKV18317.1	-6.1	46.67	-	-	-
GI:425777761	EKV15917.1	-5.9	86.23	PEXP_104370	-4.56	1.23E-12
GI:425767950	EKV06500.1	-5.8	96.39	PEXP_098120	-3.81	6.07E-10
GI:425780304	EKV18314.1	-5.8	47.35	-	-	-
GI:425770152	EKV08625.1	-5.7	96.02	PEXP_057120	-3.38	9.46E-12
GI:425772498	EKV10899.1	-5.7	86.47	PEXP_096890	-8.24	7.29E-16
GI:425765867	EKV04512.1	-5.6	96.91	PEXP_098720	-1.92	8.61E-07
GI:425777762	EKV15918.1	-5.6	91.55	PEXP_104360	-1.79	1.25E-10
GI:425770173	EKV08646.1	-5.5	97.08	PEXP_056860	-4.96	1.58E-12
GI:425771697	EKV10134.1	-5.5	89.82	PEXP_046360	-7.73	5.74E-14
GI:425770151	EKV08624.1	-5.5	93.64	PEXP_057120	-3.38	9.46E-12
GI:425775516	EKV13783.1	-5.4	96.38	PEXP_049220	-7.25	2.76E-13
GI:425766389	EKV05002.1	-5.4	94.81	PEXP_017260	-6.11	2.85E-14
GI:425780925	EKV18916.1	-5.4	-	-	-	-
GI:425768899	EKV07410.1	-5.3	74.59	PEXP_078920	-8.16	8.83E-16
GI:425781235	EKV19211.1	-5.3	63.40	PEXP_041860	-7.50	7.81E-14
GI:425780308	EKV18318.1	-5.3	89.77	PEXP_014270	-5.36	5.20E-10
GI:425773477	EKV11830.1	-5.2	92.33	PEXP_108160	-5.54	7.73E-14
GI:425774331	EKV12639.1	-5.1	79.23	PEXP_086170	-6.03	2.26E-14
GI:425778756	EKV16861.1	-5.1	88.37	PEXP_041050	-5.32	1.39E-09
GI:425770554	EKV09023.1	-5.1	80.10	PEXP_057440	-4.27	2.05E-12
GI:425772522	EKV10923.1	-5.0	97.00	PEXP_097190	-6.57	9.55E-13
GI:425769933	EKV08411.1	-4.9	85.80	PEXP_052780	-2.45	2.26E-08
GI:425777781	EKV15937.1	-4.9	96.22	PEXP_104170	ns	-
GI:425777125	EKV15315.1	-4.9	93.23	PEXP_066390	-5.00	2.02E-12
GI:425770752	EKV09216.1	-4.9	96.82	PEXP_004290	ns	-

GI:425775661	EKV13918.1	-4.9	91.64	PEXP_005290	-2.08	5.13E-10
GI:425769850	EKV08332.1	-4.9	71.97	PEX1_062950	-1.68	1.46E-08
GI:425772518	EKV10919.1	-4.8	92.96	PEXP_097110	-6.57	1.58E-12
GI:425777151	EKV15335.1	-4.7	87.87	PEXP_053700	ns	-
GI:425781931	EKV19865.1	-4.7	96.23	PEXP_018520	-7.46	1.93E-15
GI:425770818	EKV09278.1	-4.7	77.76	PEXP_031660	-5.95	9.64E-12
GI:425778273	EKV16412.1	-4.7	84.14	PEXP_025000	-8.34	6.10E-16
GI:425781017	EKV18999.1	-4.6	93.04	PEXP_072860	-1.48	3.78E-06
GI:425772484	EKV10885.1	-4.6	96.87	PEXP_096630	-12.2	6.41E-17
GI:425768595	EKV07113.1	-4.4	93.65	PEXP_077410	-3.82	1.02E-10
GI:425769499	EKV07991.1	-4.4	95.13	PEXP_027120	ns	-
GI:425767944	EKV06494.1	-4.4	55.56	PEXP_017960	ns	-
GI:425777763	EKV15919.1	-4.3	93.11	PEXP_104360	-1.79	1.25E-10
GI:425777780	EKV15936.1	-4.3	91.90	PEXP_104180	ns	-
GI:425774443	EKV12750.1	-4.2	75.84	PEXP_015010	-9.46	1.59E-15
GI:425775660	EKV13917.1	-4.2	74.88	PEXP_005280	ns	-
GI:425775572	EKV13831.1	-4.2	89.60	PEXP_109570	2.13	1.18E-08
GI:425779317	EKV17384.1	-4.2	99.16	PEXP_006710	-1.94	1.67E-10
GI:425765863	EKV04508.1	-4.2	98.94	PEXP_098670	-6.02	3.39E-13
GI:425765896	EKV04537.1	-4.1	98.09	PEXP_011080	-1.75	1.53E-09
GI:425769807	EKV08289.1	-4.1	81.42	PEXP_053340	-5.41	6.44E-13
GI:425775463	EKV13732.1	-4.1	93.61	PEXP_101250	-1.89	2.97E-10
GI:425772050	EKV10476.1	-4.0	97.94	PEXP_043320	-5.62	5.94E-13
GI:425770280	EKV08753.1	-4.0	94.29	PEXP_050580	1.29	6.05E-06
GI:425773857	EKV12182.1	-4.0	91.77	PEXP_011880	1.37	3.96E-04
GI:425770281	EKV08754.1	-4.0	94.50	PEXP_050600	1.14	4.60E-07
GI:425766239	EKV04863.1	-4.0	94.21	PEXP_094000	-5.22	4.79E-13
GI:425770817	EKV09277.1	-3.9	92.31	PEXP_031670	-7.8	3.76E-15
GI:425781867	EKV19804.1	-3.8	91.75	PEXP_098500	-1.55	1.81E-06

GI:425768068	EKV06612.1	-3.7	90.29	PEXP_054900	-9.62	7.29E-16
GI:425772896	EKV11276.1	-3.7	91.82	PEXP_029020	-3.62	4.07E-11
GI:425773464	EKV11817.1	-3.6	78.31	PEXP_108000	ns	-
GI:425779318	EKV17385.1	-3.6	95.01	PEXP_006700	-4.51	5.43E-13
GI:425777478	EKV15650.1	-3.6	92.00	PEXP_060200	-2.05	2.27E-08
GI:425769811	EKV08293.1	-3.6	95.65	PEXP_053400	-5.65	7.21E-04
GI:425773376	EKV11732.1	-3.5	92.58	PEXP_108370	-5.87	1.67E-13
GI:425767550	EKV06119.1	-3.5	95.95	PEXP_025390	-7.18	8.81E-05
GI:425775389	EKV13661.1	-3.5	95.48	PEXP_101070	-2.2	1.54E-08
GI:425767941	EKV06491.1	-3.5	96.22	PEXP_017870	ns	-
GI:425775318	EKV13596.1	-3.3	93.79	PEXP_048530	-10.7	6.41E-17
GI:425766258	EKV04882.1	-3.3	93.22	PEXP_103160	-7.8	1.41E-16
GI:425771518	EKV09959.1	-3.3	96.73	PEXP_045340	-2.46	6.74E-10
GI:425774860	EKV13155.1	-3.2	94.79	PEXP_013330	-1.13	4.40E-05
GI:425770183	EKV08656.1	-3.2	94.21	PEXP_056750	-2.05	1.35E-07
GI:425771298	EKV09744.1	-3.2	ns	-	-	-
GI:425765581	EKV04252.1	-3.1	84.34	PEXP_002300	1.01	4.37E-02
GI:425768542	EKV07063.1	-3.1	92.55	PEXP_039690	ns	-
GI:425766255	EKV04879.1	-3.1	96.27	PEXP_103220	-2.74	3.12E-04
GI:425770893	EKV09353.1	-3.0	95.65	PEXP_030880	ns	-
GI:425768940	EKV07451.1	-3.0	91.37	PEXP_078340	-5.64	7.41E-13
GI:425775292	EKV13570.1	-3.0	83.98	PEXP_048770	ns	-
GI:425766211	EKV04835.1	-3.0	96.32	PEXP_094330	ns	-
GI:425772975	EKV11353.1	-3.0	95.79	PEXP_029600	-6.06	1.28E-05

[1]PdΔ*brlA* = *Penicillium digitatum* null mutant Δ*brlA* strain. WT = wild type *Penicillium digitatum* strain

[2]PeΔ*brlA* = *Penicillium expansum* null mutant Δ*brlA* strain. WT = wild type *Penicillium expansum* strain

[*] These data are extracted from Wang *et al*. [38]

Supplementary Table 5. *Penicillium expansum* genes orthologous to genes significantly up-regulated (Log_2 fold change > 2) in *Penicillium digitatum* PdΔ*brlA* strain [38].

Penicillium digitatum PHI26 strain Gene ID	*Penicillium digitatum* GenBank Accession number	Log_2 Fold Change PdΔ*brlA* vs WT[1*]	% Positives	*Penicillium expansum* strain d1 Gene ID	Log_2 fold change PeΔ*brlA* vs WT[2]	Adjusted *p*-value
GI:425767861	EKV06414.1	2.0	96.04	PEXP_023250	ns	-
GI:425770858	EKV09318.1	2.0	89.14	PEXP_031240	ns	-
GI:425766441	EKV05051.1	2.0	79.37	PEXP_016660	ns	-
GI:425774248	EKV12561.1	2.0	96.55	PEXP_041510	ns	-
GI:425776778	EKV14982.1	2.0	95.27	PEXP_105890	1.33	7.88E-05
GI:425781020	EKV19002.1	2.1	ns	-	-	-
GI:425774460	EKV12767.1	2.1	90.71	PEXP_015220	-1.20	4.62E-03
GI:425772900	EKV11280.1	2.1	90.98	PEXP_028970	ns	-
GI:425765571	EKV04242.1	2.1	98.53	PEXP_002430	3.30	4.21E-10
GI:425766130	EKV04757.1	2.2	96.62	PEXP_080820	2.67	1.27E-08
GI:425767714	EKV06280.1	2.2	92.37	PEXP_068990	-2.59	8.77E-09
GI:425777056	EKV15250.1	2.2	95.53	PEXP_066110	ns	-
GI:425770829	EKV09289.1	2.3	86.38	PEXP_031560	1.13	8.19E-05
GI:425768269	EKV06797.1	2.3	80.59	PEXP_062990	2.63	8.79E-10
GI:425767408	EKV05982.1	2.3	42.00	-	-	-
GI:425771110	EKV09564.1	2.3	92.49	PEXP_106040	ns	-
GI:425776352	EKV14572.1	2.4	97.15	PEXP_021430	1.86	3.59E-08
GI:425771668	EKV10105.1	2.4	76.45	PEXP_061820	ns	-
GI:425770387	EKV08860.1	2.5	88.07	PEXP_051690	ns	-
GI:425769485	EKV07977.1	2.5	99.02	PEXP_026950	ns	-
GI:425773099	EKV11471.1	2.6	94.55	PEXP_000520	2.17	1.09E-07
GI:425776932	EKV15129.1	2.6	93.18	PEXP_065730	-1.46	1.78E-06
GI:425773150	EKV11520.1	2.6	85.53	PEXP_099440	ns	-
GI:425765550	EKV04227.1	2.7	43.79	-	-	-
GI:425773151	EKV11521.1	2.7	95.00	PEXP_099450	ns	-
GI:425767405	EKV05979.1	2.7	ns	-	-	-
GI:425776933	EKV15130.1	2.7	97.41	PEXP_065690	1.26	7.42E-06

GI:425779566	EKV17613.1	2.7	95.06	PEXP_054530	ns	-
GI:425772465	EKV10866.1	2.8	89.10	PEXP_096290	-2.51	4.85E-08
GI:425765551	EKV04228.1	2.8	47.10	-	-	-
GI:425771655	EKV10092.1	2.9	94.68	PEXP_061690	ns	-
GI:425771656	EKV10093.1	3.1	92.64	PEXP_061700	2.53	9.23E-08
GI:425777193	EKV15377.1	3.2	94.44	PEXP_069630	1.38	1.89E-04
GI:425765774	EKV04422.1	3.3	87.93	PEXP_099150	1.36	3.45E-08
GI:425777197	EKV15381.1	3.4	97.60	PEXP_070500	ns	-
GI:425767246	EKV05820.1	3.8	84.83	PEXP_067100	1.38	1.94E-05
GI:425776500	EKV14717.1	3.9	89.10	PEXP_021090	ns	-
GI:425781626	EKV19580.1	3.9	52.24	-	-	-
GI:425768677	EKV07195.1	4.3	71.58	PEXP_038390	-2.11	5.06E-05
GI:425781865	EKV19802.1	4.3	92.66	PEXP_098360	0.91	2.41E-03
GI:425777310	EKV15491.1	4.5	97.03	PEXP_037440	ns	-
GI:425780864	EKV18860.1	4.5	95.00	PEXP_071760	ns	-
GI:425779404	EKV17468.1	4.5	82.97	PEXP_019000	ns	-
GI:425775638	EKV13895.1	4.6	67.95	PEXP_102300	ns	-
GI:425770813	EKV09273.1	4.8	96.50	PEXP_031710	ns	-
GI:425781957	EKV19891.1	6.3	95.43	PEXP_018170	ns	-

[1]PdΔ*brlA* = *Penicillium digitatum* null mutant Δ*brlA* strain, WT = wild type *Penicillium digitatum* strain

[2]PeΔ*brlA* = *Penicillium expansum* null mutant Δ*brlA* strain, WT = wild type *Penicillium expansum* strain

[*] These data are extracted from Wang *et al*. [38]

Supplementary Table 6. Eighteen orthologous genes similarly regulated to *wetA* in *Penicillium rubens* Δ*brlA* [49] and *Penicillium expansum* Δ*brlA*.

Penicillium expansum strain d1 gene ID	*Penicillium rubens* strain * Wisconsin 54-1255 gene ID	% Identity	Log_2 fold change PeΔ*brlA* vs WT	Adjusted *p*-value
PEXP_022650	Pc12g00920	94.9	-5.87	6.90E-13
PEXP_052290	Pc12g14760	85.8	-3.79	3.17E-09
PEXP_041930	Pc13g15970	54.0	-3.42	3.41E-08
PEXP_056810	Pc18g01100	73.7	-4.35	2.83E-12
PEXP_084350	Pc19g00410	64.4	-2.12	1.90E-07
PEXP_096560	Pc19g00420	92.5	-7.51	4.58E-09
PEXP_096550	Pc20g06330	88.5	-6.75	3.06E-08
PEXP_037140	Pc21g06360	86.6	-7.61	7.77E-14
PEXP_028230	Pc21g12140	88.0	-3.90	2.87E-13
PEXP_030380	Pc21g15150	83.6	-2.17	6.41E-10
PEXP_096630	Pc21g16000	94.2	-12.20	6.41E-17
PEXP_097170	Pc21g16420	80.0	-8.64	1.31E-15
PEXP_097180	Pc21g16430	95.2	-8.34	1.13E-13
PEXP_097190	Pc21g16440	90.7	-6.57	9.55E-13
PEXP_077410	Pc22g03220	94.0	-3.82	1.02E-10
PEXP_076870	Pc22g06890	86.6	-0.99	2.35E-04
PEXP_091180	Pc22g26950	62.3	-4.38	6.23E-07
PEXP_018490	Pc20g10220	82.6	-10.7	9.61E-16

*The Wisconsin 54-1255 strain was formerly identified as *Penicillium chrysogenum*.

Supplementary Table 7. Fifty-nine orthologous genes similarly down-regulated to *abaA* in *Penicillium rubens* [49] Δ*brlA* and *Penicillium expansum* Δ*brlA*.

Penicillium expansum strain d1 gene ID	*Penicillium rubens* strain * Wisconsin 54-1255 gene ID	% Identity	Log_2 fold change PeΔ*brlA* vs WT	Adjusted *p*-value
PEXP_023320	Pc12g00010	83.2	-5.43	7.80E-14
PEXP_074900	Pc12g03260	74.2	-7.92	1.14E-14
PEXP_070580	Pc12g08750	86.5	-4.10	6.07E-10
PEXP_025850	Pc12g12350	90.8	1.49	9.90E+05
PEXP_084240	Pc12g08800	82.9	-4.15	8.76E-12
PEXP_053400	Pc12g13430	89.4	-5.65	7.21E-14
PEXP_052120	Pc12g14960	78.5	-10.10	6.09E-17
PEXP_104370	Pc13g06200	86.1	-4.56	1.23E-12
PEXP_022150	Pc13g06290	77.7	-3.61	2.72E-12
PEXP_006710	Pc13g08700	81.2	-1.94	1.67E-10
PEXP_025390	Pc13g13450	90.9	-7.18	8.01E-15
PEXP_025000	Pc13g13920	69.1	-8.34	6.10E-16
PEXP_000200	Pc14g00160	95.7	-5.57	3.94E-13
PEXP_074520	Pc15g00470	76.6	-1.68	4.01E-09
PEXP_054900	Pc16g00180	87.7	-9.62	7.29E-16
PEXP_085800	Pc16g02410	88.2	-2.51	5.36E-11
PEXP_086170	Pc16g02800	61.5	-6.03	2.26E-04
PEXP_006020	Pc16g04550	79.9	-7.01	7.37E-14
PEXP_015010	Pc16g04840	61.4	-9.46	1.59E-15
PEXP_020090	Pc16g08460	97.7	-3.17	2.09E-09
PEXP_029020	Pc16g09610	93.0	-3.62	4.07E-11
PEXP_001060	Pc16g10900	84.3	-9.51	1.43E-16
PEXP_000580	Pc16g11310	77.5	-6.21	1.49E-13
PEXP_043320	Pc16g13260	88.7	-5.62	5.94E-13
PEXP_056860	Pc18g01150	92.4	-4.96	1.58R-12
PEXP_017120	Pc18g01410	88.7	-3.38	9.46E-12
PEXP_092210	Pc18g02600	90.2	-4.28	1.47E-13
PEXP_031660	Pc18g04570	66.0	-5.95	9.64E-12
PEXP_031670	Pc18g04580	93.5	-7.80	3.76E-15
PEXP_016810	Pc20g01320	91.6	-5.75	3.58E-14
PEXP_073050	Pc20g02080	89.9	-1.49	6.57E-07
PEXP_072230	Pc20g03190	87.2	-3.22	6.03E-12
PEXP_018550	Pc20g10170	87.3	-4.36	2.05E-12
PEXP_063090	Pc20g10270	87.9	-2.75	4.31E-09
PEXP_011470	Pc20g13710	67.5	-7.65	1.10E-14
PEXP_010960	Pc20g14200	92.1	-1.17	5.06E-06
PEXP_088520	Pc21g02590	72.0	-6.66	1.17E-13
PEXP_109240	Pc21g08640	88.9	-1.39	1.54E-08

PEXP_108160	Pc21g09810	81.9	-5.54	1.73E-14
PEXP_040480	Pc21g13950	86.3	-1.57	1.56E-07
PEXP_096890	Pc21g16130	68.2	-8.24	7.29E-16
PEXP_097110	pc21g16380	79.2	-6.57	1.58E-12
PEXP_020490	Pc21g18350	76.3	-11.40	1.43E-16
PEXP_020680	Pc21g18530	81.7	-2.97	2.24E-11
PEXP_044610	Pc21g20790	70.7	-6.44	1.95E-13
PEXP_078340	Pc22g04080	76.8	-5.64	7.41E-13
PEXP_078920	Pc22g04640	92.6	-8.16	8.83E-16
PEXP_102970	Pc22g07130	93.1	-2.95	3.93E-09
PEXP_103160	Pc22g07470	89.2	-7.80	1.41E-16
PEXP_103170	Pc22g07480	82.1	-1.43	9.53E-08
PEXP_094000	Pc22g07760	83.9	-5.22	4.79E-13
PEXP_095030	Pc22g08820	54.7	-3.13	2.08E-11
PEXP_013250	Pc22g12240	94.2	-1.08	2.29E-05
PEXP_062290	Pc22g14290	87.8	-13.00	1.07E-18
PEXP_062310	Pc22g14300	76.3	-10.90	7.29E-16
PEXP_062520	Pc22g14640	89.8	-6.55	3.32E-14
PEXP_033790	Pc22g17040	61.3	-8.30	8.53E-16
PEXP_065060	Pc22g22050	91.2	-1.31	4.34E-08
PEXP_066390	Pc22g23660	90.3	-5.00	2.02E-12

*The Wisconsin 54-1255 strain was formerly identified as *Penicillium chrysogenum*.

Construction and Characterization of the null mutant Pe*ΔbrlA* strain

Creation of a Linear Transformation Cassette using Double-Joint PCR

For the creation of the null mutant Pe*ΔbrlA* strain, a strategy was applied to eliminate the *brlA* gene (gene coding for a master transcriptor factor indispensable to conidiogenesis) by replacing the coding region with the hygromycin selection marker in *Penicillium expansum* wild type NRRL 35695 (WT) strain. Specific primers of the *brlA* gene were designed from the *P. expansum* strain d1, sequence deposited in GenBank (PEXP_049260) and all primers used in this study are listed in Table S8. The Phusion High-Fidelity DNA Polymerase (Thermo Fisher Scientific, Illkirch, France) was used for all PCR amplifications.

The 5' upstream and 3' downstream flanking regions served as complementary sequences to allow homologous recombination at the locus of interest. The fragments necessary for the construction of the gene disruption marker were amplified by PCR and assembled using double-joint PCR [96, 97]. Shortly, a 1,147 bp DNA fragment that matches the promoter and the 5' untranslated region of the *brlA* gene and a 1,080 bp fragment that matches the 3' untranslated region of the *brlA* gene were amplified from the genomic DNA of the WT strain using two pairs of primers, 5fdebBrlAPex/ DebBrlAPex1Rhygro and FinBrlAPex1Fhygro/ 3rfinBrlAPex, respectively. The hphF1 / hphR1 primer pair was used to amplify a 4,107 bp fragment of pAN 7.1 plasmid (access number Z32698), which contains the hygromycin (*hph*) resistance gene [97]. The underlined sequences in the DebBrlAPex1Rhygro and FinBrlAPex1Fhygro primers were added at their 5' and 3' ends (Table S8). These sequences are identical to the extremities of the PCR product generated by the hphF1 / hphR1 primers, and served to promote binding between PCR products. The PCR conditions for the amplification of the 5' junction and 3' junction were as follows: denaturation at 98 °C for 45 s, followed by 40 denaturation cycles at 98 °C for 45 s, annealing at 61 °C for 45 s and extension at 72 °C for 1 min and 15 s. A final extension step at 72 °C for 10 min was performed. The PCR conditions for the amplification of the hygromycin resistance marker were the same as those described above, except for the annealing temperature (62 °C) and the cycle extension, which was performed for 2 min. The amplicons were purified using a GenElute™PCR Clean-Up Kit (Merck KGaA, Darmstadt, Germany), as described in the procedure specified by the manufacturer.

The three fragments were assembled by double-joint PCR, using a fragment number ratio of 1:2:1 (flank 5': marker: flank 3') according to the procedure described by Lim *et al*. [97] with slight modifications. Briefly, in the first step of the PCR we used the three amplicons previously obtained as matrices and a primerless PCR reaction is achieved, resulting in the union of the two flanking regions, the selection marker, the single-union product and the double-union product (Figure S5). The amplification conditions for this first step were as follows: denaturation at 98 °C for 5 min, followed by 12 denaturation cycles at 98 °C for 45 s, annealing at 60 °C for 2 min and extension at 64 °C for 3 min. A final extension step at 64 °C for 5 min was performed. For the second step of the procedure, the dBrlA-NestedF/dBrlA-NestedR nested primers were used, amplifying only the double-union product. For the amplification conditions a temperature gradient was followed, starting with denaturation at 98 °C for 5 min, followed by a cycle of denaturation at 98 °C for 45 s, annealing at 65 °C for 30 s and extension at 68 °C for 3 min. The annealing temperature decreased by 0.5 °C/cycle to 62 °C, followed by 24 denaturation cycles at 98 °C for 45 s, annealing at 62 °C for 30 s and extension at 68 °C for 3 min. A final extension step at 64 °C for 5 min was performed.

The final PCR product (6,292 bp), containing the hygromycin resistance marker fused to the flanking regions of the *brlA* gene, was used to transform protoplasts of *P. expansum* according to the methodology described by Snini *et al*. [22].

Supplementary Table 8. Primers used in the construction and the validation of the null mutant PeΔ*brlA* strain.

Primer	Sequence 5′ → 3′
5fdebBrlAPex	GTAACGCCAGGGTTTTCCCAGTCACGACG
DebBrlAPex1Rhygro[1]	GAGCCTGTGTGTAGAGATACAAGGGAATTCTGACCCTTGGCTGTAAAGACTGGTAGG
FinBrlAPex1Fhygro[1]	GTGTAAGCGCCCACTCCACATCTCCACTCGCGTGGCCTTCATTGGCCTTTATTGATAC
3rfinBrlAPex	GCGGATAACAATTTCACACAGGAAACAGC
hphF1	GAATTCCCTTGTATCTCTACACACAGGCTC
hphR1	CGAGTGGAGATGTGGAGTGGGCG
dBrlA-NestedF	GTTGCGGCCGGAACCTTGGAACC
dBrlA-NestedR	CCAGAGATGTCTGAGCTCCAATCGC
dBrlA-geneF	GGCTTCCCCTGGGTGGCAC
dBrlA-geneR	GCCATCGACGCTCAGCTCGC
Hyg5′1 R	CTTGACACCGCTCCGTCCTCC
Hyg3′1 F	GGGTTTACCTCTTCCAGATACAGCTC
dBrlA-5′F	GGAGTGTGGGAGATCAGGGTCG
dBrlA -3′R	CGGTGTAGCGGTGGACTCTGG
GWBrlA3′1[2]	CTTCACCGCGGCTATATGTATCGTTCAACC
GWBrlA3′2[2]	GGAACACAGTGCCCCGAATACGCACTG
Pexp_brlA Forward[3]	CCTCGATGCCTCAATACA
Pexp_brlA Reverse[3]	GTAAAGATTGGACGAGACAAG
Pexp_btub Forward[3]	TGAACGTCTACTTCAACCATGCC
Pexp_btub Reverse[3]	CCAAATCGACGAGAACGG

[1]The underlined sequences were added at the 5' end and 3′ end were identical to the extremities of the PCR product generated by primers hphF1/hphR1.

[2]Primers used in the validation by genome walking.

[3]Primers used in the validation by qPCR.

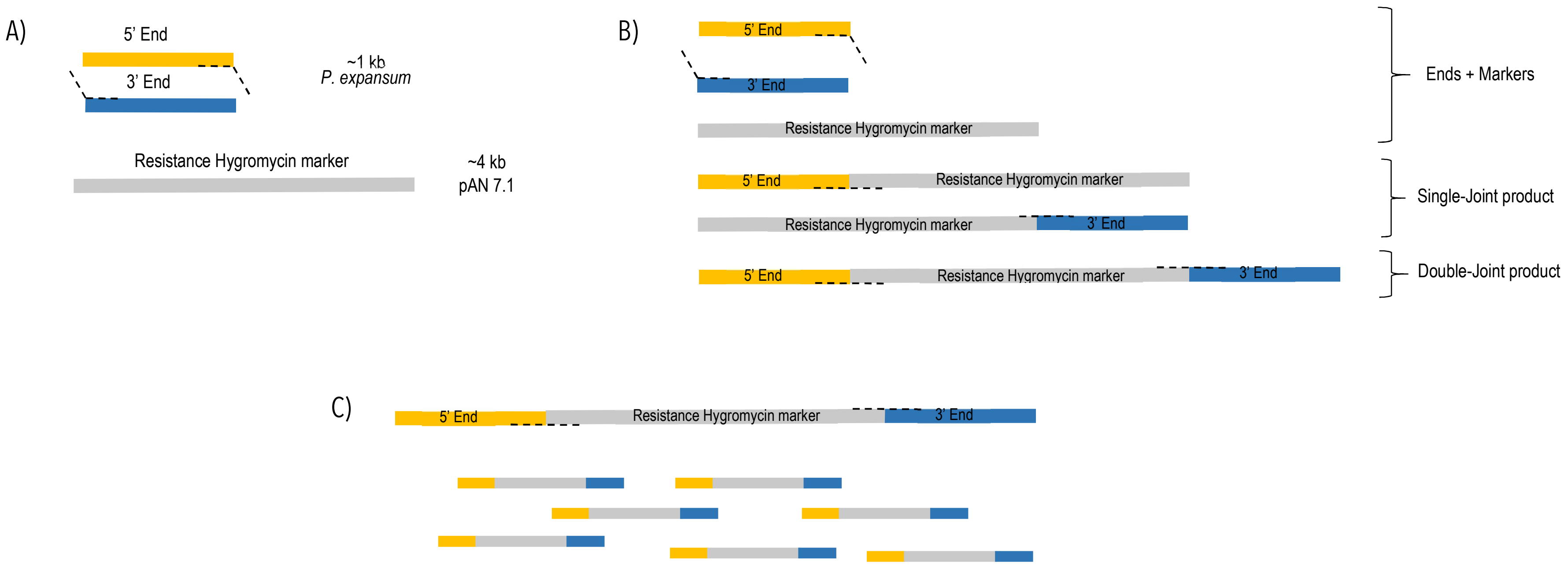

Supplementary Figure 5. Double-joint PCR reaction [97]. A) PCR reaction for amplification of flanking regions 5 'upstream and 3' downstream of the *brlA* gene from *Penicillium expansum* and amplification of the hygromycin marker from the pAN7.1 plasmid. B) Primerless PCR reaction using the three previously obtained amplicons as matrices. C) Nested PCR reaction for amplification only of the double union product.

Validation of the Null Mutant PeΔbrlA strain

Polymerase Chain Reaction (PCR) and Genome Walking

To confirm the insertion of the hygromycin resistance marker at the *brlA* locus in the transformant strains, a PCR reaction and an enzymatic digestion by genome walking were achieved. First, genomic DNA was extracted according to a previously published method [114] and used to screen the obtained transformants by PCR to detect the replacement between *brlA* and *hph*. The transformants were screened using the dBrlA-geneF/dBrlA-geneR specific primers, according to the PCR conditions: 35 denaturation cycles at 98 °C for 45 s, annealing at 62 °C for 45 s and extension at 72 °C for 1 min and 30 s. A final extension step at 72 °C for 10 min was performed. The *Taq* DNA Polymerase (Thermo Fisher Scientific) was used. Next, PCR amplifications with the dBrlA-5'F/Hyg5'1R and Hyg3'1F/ dBrlA-3'R hygromycin-specific primers were carried out on the selected null mutants. The dBrlA-5'F and dBrlA-3'R primers are located upstream and downstream of the 5' and 3' junctions of the disruption cassette, respectively, and the Hyg5'1R /Hyg3'1F primer pairs are located inside the hygromycin marker. The following PCR conditions were used: 35 denaturation cycles at 98 °C for 45 s, annealing at 60 °C for 45 s and extension at 72 °C for 2 min. A final extension step at 72 °C for 10 min was performed. The Phusion High-Fidelity DNA Polymerase (Thermo Fisher Scientific) was used.

The transformants were also verified by genome walking (GenomeWalker Universal Kit, Takara Bio Europe SAS, Saint-Germain-en-Laye, France), a simple method for finding unknown sequences adjacent to a known genomic DNA sequence. The use of this technique enabled us to verify that the cassette was inserted only once at the *brlA* locus, i.e. that it is present in a single copy in the genome of *P. expansum*. Following the protocol recommended by the manufacturer, the first step consists in constructing groups of uncloned adaptor-ligated genomic DNA fragments, referred to as "libraries". Briefly, aliquots of 2.5 µg of genomic DNA were digested overnight using the restriction enzymes EcoRV and PvuII. Each batch of digested genomic DNA was then ligated to the GenomeWalker adaptors. After the libraries were constructed, amplification was carried out by specific PCR. The first or primary PCR used the adaptor primer (AP1) provided in the kit and an external gene-specific primer (GSP1): GWBrlA3'1, which was designed from the 3' end sequence of the disruption cassette. For PCR conditions, the two-step cycle parameters were as followed: first, seven denaturation cycles at 94 °C for 25 s and annealing/extension at 72 °C for 3 min, then 32 denaturation cycles at 94 °C for 25 s and extension at 67 °C for 3 min. A final step at 67 °C for seven min was performed. The amplification product obtained from this PCR was diluted (1:50) and used as a template of a second (or nested) PCR. Primers, the nested adaptor primer (AP2) and GWBrlA3'2 (GSP2) were used. For PCR conditions, the two-step cycle parameters were as followed: first, five denaturation cycles at 94 °C for 25 s and annealing/extension at 72 °C for 3 min, then 20 denaturation cycles at 94 °C for 25 s and extension at 67 °C for 3 min. A final step at 67 °C for seven min was performed. The Advantage 2 Polymerase Mix (Takara Bio Europe SAS) was used. The sequences of the primers used are shown in Table S8. Subsequently, the fragments generated by the nested PCR were purified using a GenElute™PCR Clean-Up Kit (Merck KGaA) and two sequencing reaction were performed using the AP2 and GWBrlA3'2 primers by the BIOfidal sequencing laboratory (Vaulx-en-Velin, France). In addition, a restriction mapping was generated from genome walking products using the restriction enzymes KpnI and BstxI for the PCR product obtained from the EcoRV library, which produced fragments of 217, 483 bp and 276, 424 bp, respectively. HindIII and BamHI enzymes were used for the PCR product of the PvuII library, which produced fragments of 298, 3,702 bp and 929, 1159, 2004 bp, respectively, in accordance with the DNA genomic sequence deposited in GenBank (JQFY01000187.1).

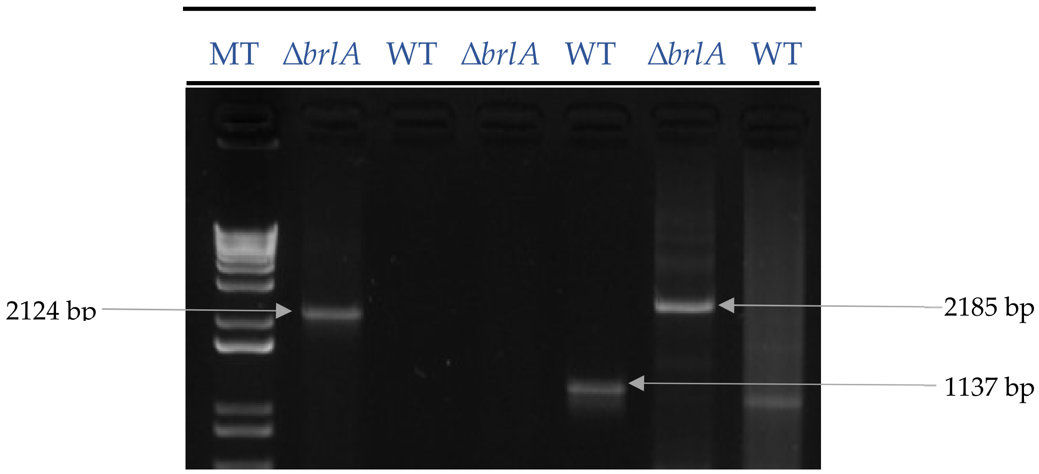

Supplementary Figure 6. PCR amplification of *Penicillium expansum* wild type NRRL 35695 (WT) and null mutant Pe*ΔbrlA* strains. Primers, lane 1 and 2: dBrlA-5′F / Hyg5′1 R, lane 3 and 4: dBrlA-geneF / dBrlA-geneR and lane 5 and 6: Hyg3′1 F / dBrlA -3′R. The presence of the disruption cassette *ΔbrlA* at the *brlA* locus was confirmed in null mutant strains amplified fragments of 2,124 bp and 2,185 bp, while no amplification was observed in the WT for PCR with the pair of primers dBrlA-5′F / Hyg5′1 R and Hyg3′1 F / dBrlA -3′R. In contrast, the presence of the *brlA* gene was not detected in the null mutant strain, the WT strain produced an amplicon of 1,137 bp. MT Lane: 1 kb plus DNA ladder.

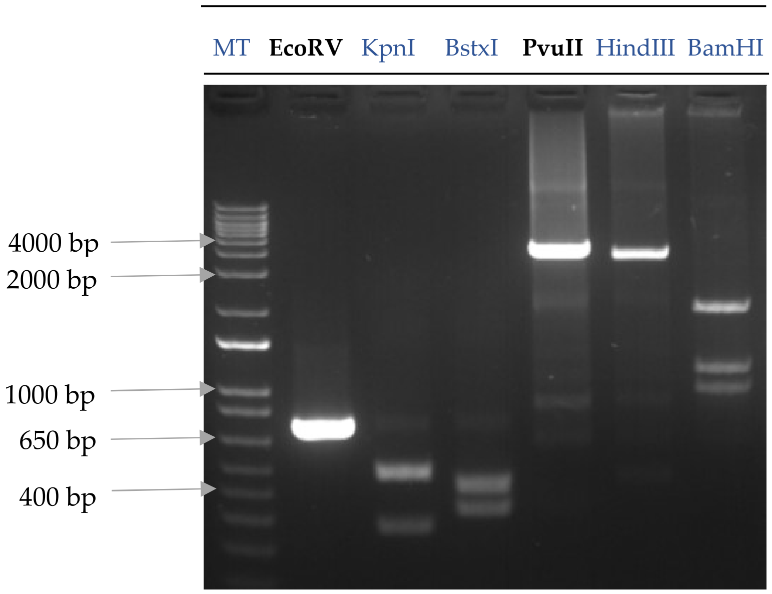

Supplementary Figure 7. Genome walking (GW) analyses of genomic DNA of *Penicillium expansum* wild type NRRL 35695 and null mutant Pe*ΔbrlA* strains. Lane 1 and 4: PCR products with the primers AP2-GWBrlA3'2 from the EcoRV (623 bp) and PvuII (4,009 bp) libraries. Lane 2 and 3: Enzymatic digestion of the EcoRV library amplicon with the enzymes KpnI (217 and 483 bp) and BstxI (276 and 424 bp) Lane 5 and 6: Enzymatic digestion of the PvuII library amplicon with the enzymes HindIII (298 and 3,702 bp) and BamHI (929 and 1,159 and 2,004 bp). MT Lane: 1 kb plus DNA ladder

Quantitative real-time PCR Validation

To investigate the gene expression of each transformant strain, a qPCR analysis was performed. The WT and null mutant PeΔ*brlA* strains were pre-cultured on MEA medium. A spore suspension was made with the WT strain and the mycelium of the null mutant strain was used as inoculation material as this strain does not produce conidia. The strains were grown in Petri dishes containing MEA covered with sterile cellophane sheets (10 µL of a 10^6 spores/mL solution for the WT strain, 5 mm^2 mycelium for the null mutant strain) and incubated at 25 °C in the dark for five days. At the end of the growth period, total RNA was isolated. From each strain, 110-120 mg of mycelium were weighed and transferred to lysing matrix D tubes (1.4 mm ceramic spheres, Thermo Fisher Scientific), to which 760 µL of lysis buffer [10 µL of β-mercaptoethanol (Applied Biosystem, Thermo Fisher Scientific) and 750 µL of RLT buffer (Rneasy mini kit, QIAGEN, Courtaboeuf, France)] were added, and were placed in liquid nitrogen. The mycelium cells homogenized in a Precellys homogenizer (Bertin Technologies, Montigny-le-Bretonneux, France) by three grindings at a speed of: 6,500 rpm during 15 s followed by 5 min incubation on ice, 6,500 rpm for 25 s and 5 min on ice and finally 6,500 rpm for 15 s. The samples were subsequently centrifuged at 16,000 g for 10 min at 4 °C. The supernatant was recovered into QIAshredder spin columns (QIAGEN) and processed according to the Rneasy mini kit (QIAGEN) protocol, detailed by Tannous *et al*. [20]. The total RNA extracted was purified using gDNA-eliminator mini-columns (QIAGEN). The RNA quality was checked by gel electrophoresis (1.2% agarose) and the concentration was determined by a NanoDrop ND1000 (Labtech, Palaiseau, France). The final RNA concentrations were around 153-255 ng/µL ($A_{260/280}$= 2.14) and 304-335 ng/µL ($A_{260-280=}$ 2.10) for the PeΔ*brlA* and WT strain respectively.

For the reverse transcription step we used a volume of 10 µl of RNA adjusted to a final concentration of 153 ng/µL and 1 µL of oligo (dT) Bys 3' Primer: (5'-GCTGTCAACGATACGCTATAACGGCATGACAGTGTTTTTTTTT-3') according to the protocol detailed by Caceres *et al*. [115].

The qPCR experiment was performed with a 7300 Real Time PCR System (Applied Biosystems, Thermo Fisher Scientific) using the three-step cycling. The primers Pexp_brlA Forward and Pexp_brlA Reverse (Table S8) were used to amplify 50-100 bp of the *brlA* target gene (PEXP_049260). The β-tubulin gene was used as the reference gene. The primers were designed and validated as described previously [115]. PCR amplification was carried out in a total volume of 25 µL, using 5 µL of cDNA template, 12.5 µL of SYBR Green PCR Master Mix (Applied Biosystems, Thermo Fisher Scientific) and 7.5 µL of primers mix (900 nM and 300 nM for the *brlA* forward/reverse primers, respectively, and 300 nM for the reference gene primers). The following PCR program was used: denaturation at 95 °C for 10 min, followed by 40 denaturation cycles at 95 °C for 15 s, annealing at 60 °C for 1 min, and a final extension step at 95 °C for 15 s, 60 °C for 1 min, 95 °C for 15 s and 60 °C for 15 s was achieved. The melting curves were analyzed with the 7300 System SDS Software (Applied Biosystems, Thermo Fisher Scientific) to confirm the specificity of the amplification. Changes in gene expression of qPCR experiments were analyzed using the $2^{-\Delta\Delta CT}$ method [116]. The Student's t-test was performed as a statistical analysis using Graph Pad 4 software (GraphPad Software, San Diego, CA, USA), a p-value < 0.05 was considered statistically significant. Four biological replicates were performed for each strain.

The RNA extraction and qPCR methodology was also used to confirm the results obtained in the microarray analysis, where the expression of all the genes of the putative chaetoglobosin cluster was evaluated. We used the RNA samples that served for the microarray analysis. The primers used are listed in Table S3.

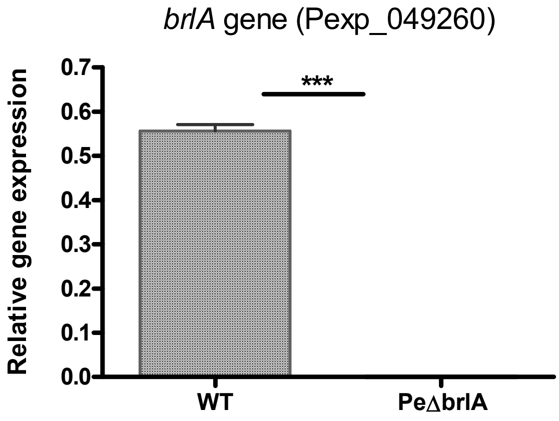

Supplementary Figure 8. Validation by quantitative real-time PCR analysis. The expression of the *brlA* gene was evaluated in *Penicillium expansum* wild type NRRL 35695 (WT) and in the null mutant Pe*ΔbrlA* strains. The Student's t-test was performed using GraphPad Software. The graph shows the mean ± standard error of the mean (SEM) from four biological replicates. *p*-value considered statistically significant: * *p*-value < 0.05; ** *p*-value < 0.01; *** *p*-value < 0.001.

References

114. Moore, G.G.; Mack, B.M.; Beltz, S.B.; Puel, O. Genome sequence of an aflatoxigenic pathogen of Argentinian peanut, *Aspergillus arachidicola*. *BMC Genom.* **2018**, *19*, 189, doi:10.1186/s12864-018-4576-2.
115. Caceres, I.; El Khoury, R.; Medina, A.; Lippi, Y.; Naylies, C.; Atoui, A.; El Khoury, A.; Oswald, I.P.; Bailly, J.-D.; Puel, O. Deciphering the anti-aflatoxinogenic properties of eugenol using a large-scale q-PCR approach. *Toxins* **2016**, *8*, 123, doi:10.3390/toxins8050123.
116. Livak, K.J.; Schmittgen, T.D. Analysis of relative gene expression data using real-time quantitative PCR and the 2-ΔΔCT method. *Methods* **2001**, *25*, 402–408.
